# Limb osteology and functional morphology of the extinct kangaroo *Dorcopsoides fossilis* (Macropodinae, Marsupialia) from Late Miocene central Australia

**DOI:** 10.1098/rsos.251591

**Published:** 2025-11-12

**Authors:** Isaac A. R. Kerr, Jasmin Hoadley, Gavin J. Prideaux, Aaron B. Camens

**Affiliations:** ^1^ College of Science and Engineering, Flinders University, Adelaide, South Australia, Australia

**Keywords:** Neogene, Miocene, fossil, Macropodidae, Dorcopsini, forest wallaby, ecomorphology

## Abstract

The ‘modern’ kangaroos and wallabies (subfamily Macropodinae) are herbivorous marsupials characterized by their bipedal hopping gait. Macropodines radiated through the Late Miocene and Pliocene (11.6–2.6 Ma) as Australia aridified. *Dorcopsoides fossilis*, known only from the Late Miocene Alcoota locality in central Australia, is the earliest undisputed macropodine. Although first described in 1967 from craniodental and postcranial fragments, it has since received minimal attention, despite the accumulation of many more specimens and the potential they hold for shedding light on kangaroo evolution. Here we describe its limb morphology and make comparisons with limb bones of extant species with various locomotory modes and habitat preferences. Though *D. fossilis* is generally similar to living low-geared hoppers, it has certain features seen in higher-geared macropodines that imply some ability to hop powerfully and efficiently. These features indicate the species was adapted to moving through open habitats, consistent with past interpretations of the Alcoota palaeoenvironment. As in modern macropodines, the pronounced size bimodality in forelimb elements of *D. fossilis* likely represents sexual dimorphism. Our results strongly reinforce the macropodine affinity of *D. fossilis*, demonstrating that the dominant features of the macropodine skeletal plan were in place well before the end of the Miocene.

## 1. Introduction

The family Macropodidae is a diverse group of marsupials that exhibits a broad array of morphologies and occupies a wide range of habitats. It includes the extant subfamilies Macropodinae (‘modern’ kangaroos and wallabies) and Lagostrophinae (one extant species, *Lagostrophus fasciatus* (Péron & Lesueur, 1807) [1], the extinct subfamily Sthenurinae (short-faced kangaroos) and several extinct stem taxa [2]. Macropodids are inferred to have originated in the Oligocene [3,4], had small body sizes, low-crowned molars and a probable quadrupedal, bounding gait [5–7]. Analysis of calcaneal morphology has suggested that the plesiomorphic macropodoids of family Balbaridae, along with basal macropodids from the Late Oligocene and Early Miocene, likely hopped bipedally, leading to the inference that bipedal hopping is the ancestral high-speed gait of all macropodoids except hypsiprymnodontids [8–10]. Driven by the spread of open habitats as central and western Australia aridified in the late Middle to Late Miocene [11,12], larger macropodine and sthenurine kangaroos emerged as the dominant terrestrial macropodoids [2,13–16]. Macropodine kangaroos, in particular, diverged from the ancestral low-geared state (i.e. inefficient hopping and low maximum speed), developing mid- and high-geared (i.e. efficient hopping and high maximum speed) forms for effective movement through sparse vegetation [2,17]. The radiation of Macropodinae and Sthenurinae is clear from the Pliocene and Pleistocene records, with the ancestors of all modern macropodine genera inferred from molecular divergence estimates to have existed by the end of the Miocene [2,18,19]. However, the patchiness of the Middle and Late Miocene record means that few macropodid species are known from this critical period in the evolution of this iconic, ecologically important group.

The mid- to late Cenozoic was a period of significant climatic change across Australia, driving sweeping changes in the Australian biota. From the Oligocene to the Early Miocene Australia’s climate was warm and much wetter than it is today, with diverse forest types [11,20,21]. Rainforest biomes in northern Australia were probably most extensive during the Early and Middle Miocene [11,22], with palaeobotanical evidence suggesting a dominance of dry woodlands with no rainforest in western and central Australia by the Middle Miocene [11,23]. From the Middle to Late Miocene (16.4–5.3 Ma), global cooling, Antarctic ice-sheet expansion, and the northward drift of the Australian continent drove an intensification of aridity across much of inland Australia [11,21,24,25]. Open, eucalypt-dominated woodlands and sclerophyllous shrublands thrived in the increasingly xeric conditions [11,26]. This environmental change coincided with the disappearance of several macropodoid lineages and the radiation of larger, more open-habitat-adapted macropodids [6,27].

Stem macropodids with fully bilophodont molars and early sthenurines occur in the Encore Local Fauna (LF) of Riversleigh, northwestern Queensland, which is estimated by stage-of-evolution biochronology to date to 12–10 Ma. There is a gap in the Australian mammal record of perhaps 2–4 million years between the Encore LF and the macropodid-bearing deposit considered to be the next youngest: the Alcoota LF of the southern Northern Territory [6]. As with the Encore LF, stage-of-evolution biochronology is the only means by which the Alcoota LF and the stratigraphically overlying Ongeva LF have been temporally constrained, and together, their taxa define the Waitean land mammal age [28]. The Early Pliocene Sunlands and Hamilton LFs of southeastern Australia lie within next-youngest window for terrestrial vertebrate sites yielding macropodoids [29–31].

The geology and taphonomy of the Alcoota deposit suggest accumulation in a fluvio-lacustrine setting marked by seasonal fluctuations in rainfall, with many of the preserved animals considered to have died as a result of drought before their remains were concentrated during one or more flooding events [32,33]. The mammalian component of the assemblage is dominated by large-bodied herbivores, including five diprotodontoids [34,35]. Two macropodids are known from Alcoota LF: *Hadronomas puckridgi* Woodburne, 1967 [34], a stem sthenurine, and *Dorcopsoides fossilis* Woodburne, 1967 [34], the earliest known macropodine.

When *Dd. fossilis* was first described it was on the basis of incomplete cranial, dental and pedal material [34]. Brief comparisons were made with morphologically similar extant species. Woodburne [34] considered *Dd. fossilis*, along with the species of *Dorcopsis* Schlegel & Müller, 1845 [36] and *Dorcopsulus* Matschie, 1916 [37], to belong to Potoroidae. However, all are today grouped in the basal macropodine tribe Dorcopsini on the basis of dental and osteological characters [2], supported by molecular data in the case of the living genera [18,19]. A single unambiguous synapomorphy links the dorcopsins, including *Dorcopsoides*: lateral constriction of the upper permanent premolar (P3) immediately anterior to the posterolingual cusp [2]. *Dorcopsis* and *Dorcopsulus* are considered sister to the remaining extant macropodines, with a divergence from *Dorcopsoides* Woodburne, 1967 [34] estimated to have occurred around 14–11 Ma, according to molecular divergence estimates [18,19,38].

Dorcopsini (‘forest wallabies’) is the only macropodid tribe absent from the modern fauna of mainland Australia, with extant members restricted to New Guinea and some adjacent islands. Of the six living species three are considered to have stable populations (*Dorcopsis muelleri* [Lesson, 1827] [39], *Dorcopsis hageni* Heller, 1897 [40] and *Dorcopsulus macleayi* [De Miklouho-Maclay, 1885] [41]), while the remainder are near-threatened (*Dorcopsulus vanheurni* [Thomas, 1922] [42]), vulnerable (*Dorcopsis luctuosa* [D’Albertis, 1874] [43]) and critically endangered (*Dorcopsis atrata* van Deusen, 1957 [44]) [45–50]. Dorcopsins are found in habitats ranging from dense upper montane rainforest to lowland tropical forest [51–53]. The group is markedly understudied and species ecologies are poorly understood; dorcopsins are considered browsers, with some species known to dig for and consume fungi [51,54,55]. Adult body masses range from 1.5 to 10 kg [52].

Whereas living dorcopsins only occur in New Guinea, dorcopsin fossils are known from three Australian localities: *Dd. fossilis* from Alcoota, *Dorcopsis* sp. indet. from Sunlands [29] and *Dorcopsis wintercookorum* Flannery *et al.*, 1992 [30] from Hamilton. *Watutia novaeguineae* Flannery, Hoch & Aplin, 1989 [56] and *Dorcopsoides buloloensis* (Plane, 1967) [57], from the late Pliocene Otibanda Formation in eastern New Guinea, have recently been considered members of Dorcopsini [58,59]. Both are represented by a small amount of craniodental material. Originally considered a member of the extinct macropodin genus *Protemnodon*, and later the now-defunct *Silvaroo* Dawson, 2004 [60], *Dd. buloloensis* was only recently recognized as a congener of *Dd. fossilis* [59]. *Dorcopsis wintercookorum* is known only from partial maxillae and isolated lower cheek teeth [30]. Consequently, there is very little understanding of the evolutionary history of dorcopsins. At present, *Dd. fossilis* is one of only two fossil dorcopsin for which postcranial elements are known. Analysis of these holds great potential for shedding light on a critical stage in the evolution of the iconic macropodine gaits.

Gait use varies markedly between different macropodoid groups and is strongly phylogenetically influenced. Some extinct macropodid groups had markedly different locomotor modes from those seen in modern kangaroos—for example, sthenurines, particularly the larger-bodied taxa, are thought to have stridden bipedally [61]. Extant macropodoids use different gaits for low- and high-speed locomotion [62–64], with the exception of the sole extant member of the plesiomorphic Hypsiprymnodontidae, *Hypsiprymnodon moschatus* Ramsay, 1875 [65], which only employs a quadrupedal bound, where both forefeet contact the substrate, more or less simultaneously, followed by both hindfeet [9]. Most living macropodoids use bipedal hopping (saltation) as their primary fast gait, where the hindlimbs make contact with the ground simultaneously and the forelimbs are held in front of the body [62]. A plesiomorphic quadrupedal fast gait, wherein all four limbs make contact with the ground at some time throughout the locomotory cycle, is used by few species [9,62,66]. All living macropodoids except *Hy. moschatus* are capable of hopping [9,62,67], including the potoroid *Potorous tridactylus* (Kerr, 1792) [68], which hops bipedally in high-speed locomotion, but has been observed to fast-bound quadrupedally under laboratory conditions for short periods [66,69].

For slow locomotion, macropodoids utilize either a quadrupedal or a ‘pentapedal’ bound. A pentapedal bound (‘punt’), wherein the tail is used as a ‘fifth limb’ for support while planting two hands on the ground and swinging the hindlimbs forward, is used for slow locomotion by all living macropodins [64]. With the exception of dorcopsins, macropodines with a crural index (tibia length divided by femur length) below 1.45 do not use pentapedal locomotion as a slow gait [64], but use a quadrupedal bound instead, as do all non-macropodine macropodoids [66]. Extant dorcopsins are unique among macropodids when moving slowly because they strongly arch the tail so that only the last few centimetres touch the ground and bear weight [62,70]. The adaptive significance of this trait is not known. An untested idea exists colloquially among New Guinean naturalists that the tail is kept predominantly off the ground in this manner due to the wet, muddy substrate in New Guinean rainforests and the need to keep the tail relatively clean and dry (I.A.R.K., personal observation).

Habitat has had a strong influence on the development of different gaits [17,62,71]. More quadrupedal macropodoids tend to live in places with dense ground cover, whereas those that employ a bipedal gait more frequently are more common in open habitats such as grasslands, shrublands and grassy woodlands [72]. A quadrupedal bound increases agility in areas of dense vegetation, while a bipedal hop is a fast and highly efficient locomotory mode across open areas [9,17,73]. Potoroids that live in more mesic environments (e.g. *Po. tridactylus*) hop less frequently than those inhabiting more arid environments (e.g. *Bettongia penicillata* [Gray, 1837] [74]), although they are still capable hoppers [75,76].

Comparison of *Dd. fossilis* with the living members of tribe Dorcopsini and extant species may provide some insight into its locomotory mode and ecomorphological adaptations. In living dorcopsins a quadrupedal bound is used interchangeably with bipedal hops for mid-speed locomotion, while a short, rapid bipedal hop is used for mid- to high-speed locomotion [62,70]. Locomotory mode diversity in macropodoids is reflected in limb bone morphology and proportions. In species that hop with greater frequency lengths of the tibia and metatarsal IV increase in relation to femur length [17]. Additionally, a greater disparity between shorter forelimb and longer hindlimb bone lengths is present in predominantly bipedal macropodoids. For example, the highly efficient, bipedal-hopping *Osphranter rufus* (Desmarest, 1822) [77] has greater disparity between forelimb and hindlimb length than the more quadrupedal *Po. tridactylus*, and the tree-kangaroo *Dendrolagus matschiei* Förster & Rothschild, 1907 [78] has no disparity between forelimb and hindlimb lengths [79]. The size and relative position of muscle attachment areas in the limbs of various species of fossil kangaroo have been used to infer adaptations to bipedal hopping, scansoriality and even bipedal striding (e.g. Janis *et al*. [61], Warburton & Prideaux [80]).

Several studies have identified features of *Dd. fossilis* that support its position as a stem macropodine and ancestral dorcopsin, though none have touched on its functional morphology. A number of studies have compared and discussed the craniodental morphology of *Dd. fossilis* (e.g. Dawson [60], Cooke [60,81]), but its postcranial skeleton is less well studied. Murray [82] compared and discussed the humerus, tibia, talus, calcaneus, cuboid, metatarsal V and pedal phalanges of *Dd. fossilis* as part of a thorough description of the postcranial skeleton of the early sthenurine *Ha. puckridgi*, and noted the following characteristics in *Dd. fossilis* that separate it from *Ha. puckridgi* and other sthenurines: a humerus lacking the small crest on the lateral epicondyle present in sthenurines; a cuboid with a narrower, more plantarly prominent lateral plantar tuberosity; a talus with a posterolateral tubercle present and a smaller scar for the calcaneo-talar ligament; and a calcaneus with a dorsoplantarly shorter dorsolateral cuboid facet and a broader plantomedial cuboid facet. As there has been only this partial comparison and no detailed description or functional examination of the postcranial morphology of the species, it is not known whether *Dd. fossilis* utilized bipedal saltation as its primary method of high-speed terrestrial locomotion in the manner of almost all living macropodines.

Undescribed postcranial material collected over the past four decades provides the opportunity to expand our knowledge of the morphology of *Dd. fossilis*. This study aims to undertake a comparative description of the postcranial skeleton and make inferences about the functional morphology of its limbs. This will inform upon the evolution of dorcopsins and the ancestral gait of macropodines.

## 2. Material and methods

### 2.1. Descriptions and comparisons

Nomenclature follows Warburton *et al*. [83], Warburton & Prideaux [80] and Kerr *et al*. [84]. Description and comparison of muscle attachment sites are informed chiefly by Warburton *et al.* [85] for the forelimb, and by Hopwood & Butterfield [86] and Warburton *et al*. [87] for the hindlimb.

Comparative specimens were accessed from the mammal collection of the South Australian Museum, Adelaide (SAMA M), the Flinders University Vertebrate Collection (FUR), the Australian National Wildlife Collection (ANWC CM), the mammals collection of the Western Australian Museum (WAM M) and as digital specimens through Ozboneviz [88]. Fossil material was accessed from the palaeontology collection of the Museum and Art Gallery of the Northern Territory (NTM P). A list of comparative specimens is available in the electronic supplementary material.

Limb elements of *Dorcopsoides fossilis* were compared with those of various living and extinct macropodids. The postcranial skeletons of the living dorcopsins *Dorcopsis luctuosa*, *Dorcopsis muelleri* and *Dorcopsulus vanheurni* were used. *Macropus fuliginosus* Desmarest, 1817 [89], *Notamacropus eugenii* (Desmarest, 1817) [89], *Petrogale xanthopus* Gray, 1855 [90] and *Thylogale billardierii* (Desmarest, 1822) [77] were used for comparison within the predominantly hopping macropodines, and *Setonix brachyurus* (Quoy & Gaimard, 1830) [91] and *Dendrolagus lumholtzi* Collett, 1884 [92] were used as terrestrial and arboreal examples, respectively, of predominantly non-hopping macropodines. *Potorous tridactylus* and *Bettongia lesueur* (Quoy & Gaimard, 1824) [93] were included to represent the plesiomorphic potoroids. Non-macropodine macropodids were included in order to establish shared plesiomorphic characteristics. *Ngamaroo archeri* Kear & Pledge, 2007 [94]from the late Oligocene Etadunna Fm. in central Australia, and *Ganguroo robustiter* Cooke *et al.*, 2015 [95] from the Middle Miocene of Riversleigh in northeastern Australia were included because they are the only two basal macropodids with published descriptions of postcranial elements (see Kear *et al*. [96], Kear & Pledge [94]). *Hadronomas puckridgi*, a sthenurine also found in the Alcoota LF, and the extant lagostrophine *Lagostrophus fasciatus* were also included in comparisons. The rarity of complete dorcopsin comparative specimens impacted comparison of the manus; the available specimen of *Du. vanheurni* has only a single metacarpal II, and the available specimens of *Do. luctuosa* and *Do. muelleri* lack fourth metacarpals.

### 2.2. Mensuration

For the purposes of comparison in skeletal proportions, body mass estimation and detection of sexual dimorphism, measurements were taken from specimens of *Dd. fossilis* and of all species listed above for inclusion in the comparative descriptions. Elements measured were major limb elements, metacarpals, metatarsals and phalanges. Measurements were taken from all sufficiently intact specimens of *Dd. fossilis* and listed in tables within the descriptions and comparisons. Specimens missing epiphyses were excluded. The complete morphometric dataset including measurements taken from comparative specimens is available in the electronic supplementary material.

### 2.3. Principal component analysis

Five calcaneal measurements (calcaneal length, head width, tuber depth, tuber width, width of talar facets) were collected and collated from the 12 complete calcanei of *Dorcopsoides fossilis* and from 16 extant comparative species. The data were transformed to remove the effect of absolute size from the analysis, using the workflow of Mein *et al.* [97]. The geometric mean of the dataset was calculated, each measurement was divided by the geometric mean, and then log transformed (log_10_). A principal component analysis (PCA) was run on this dataset using the ‘prcomp’ command in R v4.4.1 using RStudio-2024.04.2-764, with the data scaled then visualized in scatterplots of the resulting first and second principal components. This is similar to the analysis conducted by Janis *et al.* [8], which included PCA of calcaneal measurements including a single calcaneus of *Dd. fossilis* and predominantly focused on basal macropodoids. The analysis conducted for this study has a larger number of specimens of *Dd. fossilis* is more focused on macropodines, though it includes four non-macropodine macropodoids (see below).

Species in the analysis were grouped based on locomotory adaptation. This includes use of the term ‘gearing’ to describe predominantly bipedal, non-arboreal macropodoid relative locomotory efficiency and top speed, following Murray [98] and Kerr *et al.* [84]. ‘Low-geared’ macropodoids are less efficient and with a low top speed. In the PCA these were *Setonix brachyurus*, *Dorcopsis luctuosa*, *Dorcopsis muelleri*, *Dorcopsulus vanheurni*, *Thylogale billardierii*, *Potorous tridactylus* and *Bettongia lesueur*. ‘Mid-geared’ are generalized hoppers with moderate efficiency and speed. In the PCA these were represented by *Notamacropus eugenii*, *Notamacropus rufogriseus* [89], *Petrogale xanthopus* and *Lagostrophus fasciatus*. ‘High-geared’ are efficient, sustained hoppers with a high top speed, represented in the analysis by *Osphranter robustus* (Gould, 1841) [99] and *Macropus fuliginosus*. Also included were two arboreal species, *Dendrolagus bennettianus* De Vis, 1887 [100] and *Dendrolagus lumholtzi*, and the sole living obligate-quadruped macropodoid, *Hypsiprymnodon moschatus*. The dataset is available in the electronic supplementary material.

### 2.4. Body mass estimates

Body mass estimates were made using the method and dataset of Prideaux & Warburton [58]. That study constructed a dataset of body masses and skeletal measurements from 63 specimens of extant species of macropodine, including three dorcopsins (*Dorcopsis atrata*, two specimens of *Dorcopsis muelleri*, and *Dorcopsulus vanheurni*) (see [58, table 9]). These data were then log transformed (log_10_) to create predictive equations based on regression analyses (see [58, table 10]). The measurements taken were minimum femoral circumference (FC), calcaneal length (CL), tuber width (CTW) and tuber depth (CTD), with calcaneal measurements also multiplied to two- and three-dimensional variables (CL × CTD; CL × CTW; CL × CTD × CTW). In our analysis, measurements were taken from four femora and 25 calcanei of *Dorcopsoides fossilis*. The dataset with equations is available in the electronic supplementary material.

## 3. Systematic palaeontology

Class MAMMALIA Linnaeus, 1758 [101]

Order DIPROTODONTIA Owen, 1877 [102]

Suborder MACROPODIFORMES Ameghino, 1889 [103]

Superfamily MACROPODOIDEA Gray, 1821 [104]

Family MACROPODIDAE Gray, 1821 [104]

Subfamily MACROPODINAE Gray, 1821 [104]

Tribe DORCOPSINI Prideaux & Warburton, 2010 [2]

**Dorcopsoides** Woodburne, 1967 [34]

*Type species: Dorcopsoides fossilis* Woodburne, 1967

**Dorcopsoides fossilis** Woodburne, 1967

*Dorcopsoides fossilis* Woodburne, 1967: *Bull. Bur. Min. Res. Geo. Geophys.*, *Aus.*, pp. 44–81, figures 10–13, tables 5–15. See also Kerr & Prideaux [59], pp. 6–8, figures 2 and 3, tables 1 and 2.

### 3.1. Referred material

Bracketed numbers indicate the number of same elements assigned to a catalogue number when element amount is two or more.

Main Pit, Alcoota LF, Alcoota Station, Northern Territory:

NTM P6046, NTM P13739 scapula. NTM P10974, NTM P17479 humerus. NTM P6360 radius. NTM P6060, NTM P6061, NTM P10353, NTM P10659 ulna. NTM P17668 pisiform. NTM P15658 hamatum (unciform). NTM P4962 metacarpal III. NTM P6117 metacarpal IV. NTM P18032 associated partial hindlimb (distal femur, tibia, metatarsal IV). NTM P4985, NTM P6092 pelvis (innominate). NTM P18448, NTM P5585, NTM P5595, NTM P6017, NTM P6113, NTM P6118 femur. NTM P4322, NTM P5569, NTM P5576, NTM P5578, NTM P92137 tibia. NTM P4844, NTM P4848, NTM P4855, NTM P4963, NTM P15138 fibula. NTM P10953, NTM P10954, NTM P10955, NTM P10956, NTM P10957, NTM P10958, NTM P10959, NTM P10960, NTM P10961, NTM P10962, NTM P10963, NTM P10964, NTM P10965, NTM P10966, NTM P10967, NTM P10968, NTM P10969, NTM P10970 associated partial pes (distal fibular epiphysis, talus, calcaneus, cuboid, metatarsal II?, proximal phalanx II?, metatarsal III?, proximal phalanx III?, metatarsal IV, proximal phalanx IV, middle phalanx IV, distal phalanx IV, metatarsal V, proximal phalanx V, middle phalanx V, distal phalanx V, pedal sesamoid, pedal sesamoid).

NTM P4502, NTM P4617, NTM P4619, NTM P4904 (10), NTM P6011, NTM P6012, NTM P18449, NTM P18450 calcaneus. NTM P4458, NTM P4971, NTM P5778 (3), NTM P5779 (2), NTM P5786 (2), NTM P5788, NTM P5791 (2), NTM P5793, NTM P5794, NTM P5799, NTM P5789, NTM P5801 (4), NTM P8828 talus (astragalus). NTM P4504, NTM P4528 (5), NTM P5399 (4), NTM P5785 cuboid. NTM P18451, NTM P15319, NTM P16953 navicular. NTM P4415, NTM P4416, NTM P6014 ectocuneiform. NTM P6430 metatarsal II. NTM P10658 metatarsal III. NTM P4298, NTM P4525, NTM P4526, NTM P4692 (2), NTM P4699, NTM P4700, NTM P5575 (4) metatarsal IV. NTM P6081 metatarsal V. NTM P5491 (2), NTM P5493, NTM P5494 (3), NTM P5496, NTM P5513, NTM P5514, NTM P5527, NTM P5529, NTM P5564, NTM P5590 proximal pedal phalanx IV. NTM P18452, NTM P18453 middle pedal phalanx V. NTM P10879, NTM P10951 (3), NTM P18454 distal (ungual) pedal phalanx V.

Dredd locality, Alcoota LF, Alcoota Station, NT:

NTM P8711-5 ulna. NTM P8757-1 metacarpal I. NTM P5787-5, NTM P8757-6 metacarpal II. NTM P8757-4, NTM P8757-8 metacarpal IV. NTM P8754 pelvis. NTM P8768 tibia. NTM P878 (21) calcaneus. NTM P8718-1 talus. NTM P876-1, NTM P876-8 metatarsal IV. NTM P8714-49/58, NTM P8714-77, NTM P8714-78, NTM P8714-84, NTM P8714-86, NTM P8714-93, NTM P8714-91 proximal pedal phalanx IV. NTM P8762 (8) distal pedal phalanx V.

Middle Pit, Alcoota LF, Alcoota Station, NT:

NTM P8775-13 metatarsal IV.

Rochow locality, Alcoota LF, Alcoota Station, NT:

NTM P8798-4 calcaneus.

Shattered Dreams locality, Alcoota LF, Alcoota Station, NT:

NTM P4922 tibia. NTM P4847 fibula. NTM P4921 calcaneus. NTM P4920 talus. NTM P4757 (2), NTM P4758 cuboid. NTM P4405, NTM P4406 metatarsal V. NTM P4996 proximal pedal phalanx IV.

South Pit, Alcoota LF, Alcoota Station, NT:

NTM P13741 hamatum. NTM P6099 pelvis. NTM P4505 calcaneus. NTM P5808 talus. NTM P4804 (2), NTM P4805 cuboid. NTM P4711 metatarsal IV. NTM P4562 metatarsal V. NTM P4806 (2), NTM P5466, NTM P5468, NTM P5469 proximal pedal phalanx IV. NTM P4812 middle pedal phalanx IV. NTM P4813, NTM P4814 proximal pedal phalanx V.

Alcoota LF (site unknown), Alcoota Station, NT:

NTM P5577, NTM P6087, NTM P6089, NTM P6090, NTM P6091 humerus. NTM P6084, NTM P879-1, NTM P879-3, NTM P879-4 radius. NTM P6058, NTM P6059, NTM P6062, NTM P6065, NTM P6066, NTM P6068, NTM P8711-3 ulna. NTM P13486 hamatum. NTM P5572 metacarpal II. NTM P5571 (2), NTM P5573 (2), NTM P5574 metacarpal IV. NTM P6106, NTM P9292 pelvis. NTM P1007, NTM P5594, NTM P6016, NTM P6018, NTM P6019, NTM P92136, NTM P4357, NTM P4426, NTM P6020 femur. NTM P4369, NTM P5587 (2), NTM P6114, NTM P8768 tibia. NTM P4851, NTM P4853, NTM P12841, NTM P12880, NTM P92200 fibula. NTM P4621 calcaneus. NTM P5810 talus. NTM P4503, NTM P4590, NTM P4591 (10) cuboid. NTM P15323, NTM P15324 navicular. NTM P6013 (3), NTM P6015 (2) ectocuneiform. NTM P4564, NTM P4565, NTM P4566, NTM P4567, NTM P4568, NTM P8867 metatarsal IV. NTM P4580, NTM P4581, NTM P4582 metatarsal V. NTM P8855 proximal pedal phalanx IV. NTM P13634 distal pedal phalanx V.

### 3.2. Descriptions and comparisons

#### 3.2.1. Scapula

**Figure 1.**
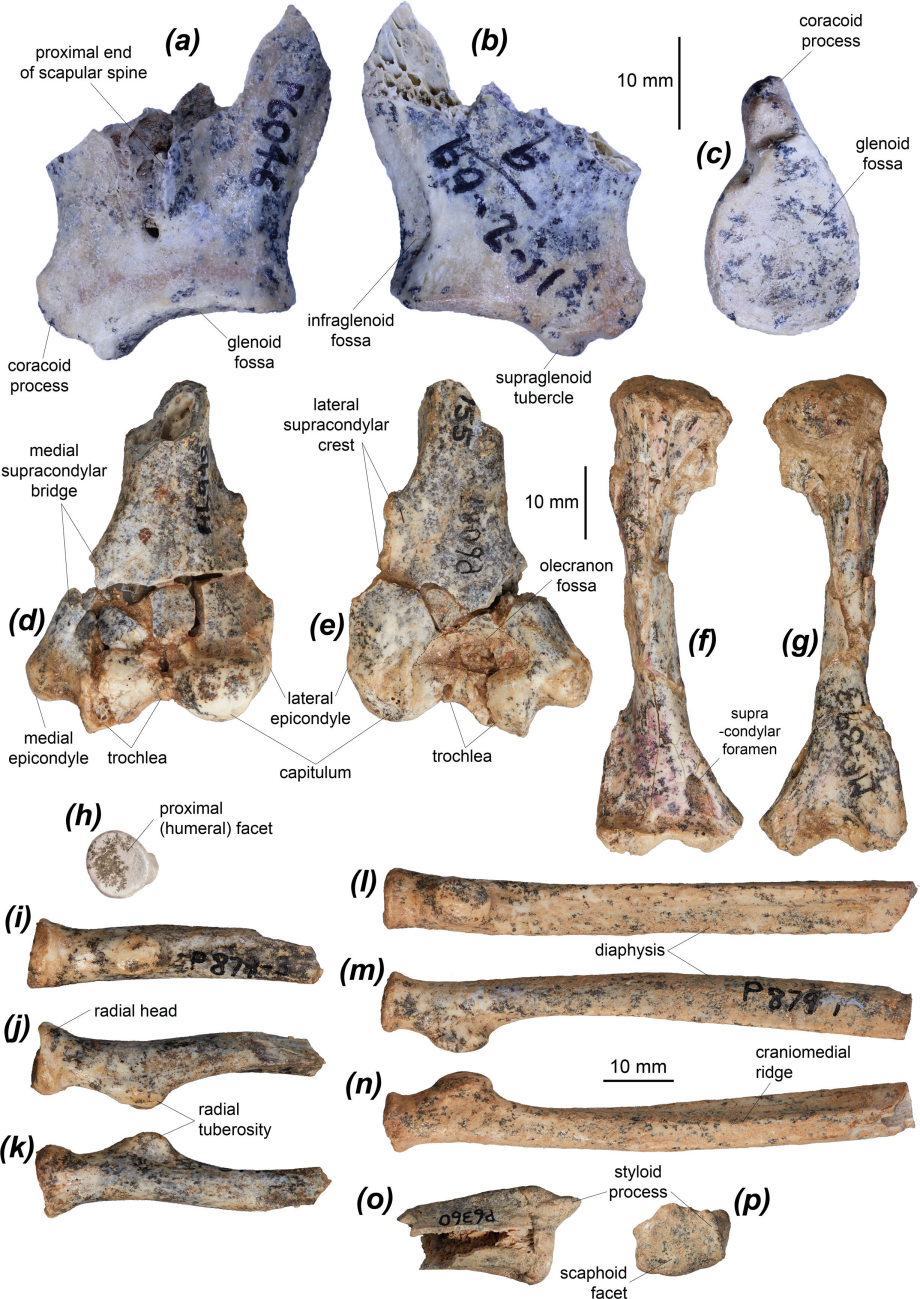
Forelimb elements of *Dorcopsoides fossilis*: (*a*–*c*) proximal (humeral) left scapular fragment NTM P6046 in (*a*) dorsolateral, (*b*) ventromedial and (*c*) proximal views; (*d,e*) distal left humeral fragment NTM P6087 in (*d*) cranial and (*e*) caudal views; (*f,g*) juvenile right humerus in (*f*) cranial and (*g*) caudal views; (*h*–*k*) proximal right radial fragment NTM P879-3 in (*h*) proximal, (*i*) caudal, (*j*) lateral and (*k*) medial views; (*l*–*n*) partial left radius NTM P879-1 in (*l*) caudal, (*m*) medial and (*n*) lateral views; and (*o,p*) distal left radial fragment NTM P6360 in (*o*) cranial and (*p*) distal views.

The scapula of *Dorcopsoides fossilis* is represented by two proximal fragments (figure 1*a–c*). Only the proximal base of the scapular spine is known (figure 1). The glenoid fossa is rounded and of moderate depth, most similar to *Thylogale billardierii*, *Petrogale xanthopus* and *Dorcopsis luctuosa*, although in *Pe. xanthopus* the fossa is slightly less elongate craniocaudally. *Notamacropus eugenii*, *Setonix brachyurus*, *Bettongia lesueur* and *Potorous tridactylus* have a more deeply concave glenoid fossa than in *Dd. fossilis*. The glenoid fossa is more rounded in lateral (humeral) view (figure 1*c*) than in *M. fuliginosus*, *No. eugenii*, *S. brachyurus*, *Dendrolagus lumholtzi*, *Hadronomas puckridgi* and *Lagostrophus fasciatus*, and is more oval/less rounded than those of *Dorcopsulus vanheurni* and *Dorcopsis muelleri*. The cranial end of the fossa is narrowed, as in all other examined species, with the supraglenoid tubercle projected cranially on the cranial margin. This tubercle is fairly small and projects only slightly laterally, separated from the small, cranially projected coracoid process by a small fossa (figure 1*b*). The coracoid process is large, more cranially situated relative to the glenoid fossa and is not prominent laterally, similar to those of *M. fuliginosus*, *No. eugenii*, *S. brachyurus* and *Do. luctuosa*. In *T. billardierii*, *Pe. xanthopus*, *De. lumholtzi*, *Ha. puckridgi*, *Ganguroo robustiter*, *B. lesueur* and *Po. tridactylus* the coracoid process protrudes laterally, more so than the supraglenoid tubercle, and is longer and curved medially in *L. fasciatus* and both potoroids. In *Du. vanheurni* and *Do. muelleri*, the supraglenoid tubercle and coracoid process are smaller and less cranially projected. The supraglenoid and coracoid are discrete, unlike in *M. fuliginosus*, *T. billardierii*, *Du. vanheurni*, *Do. muelleri* and *L. fasciatus*.

#### 3.2.2. Humerus

**Figure 2.**
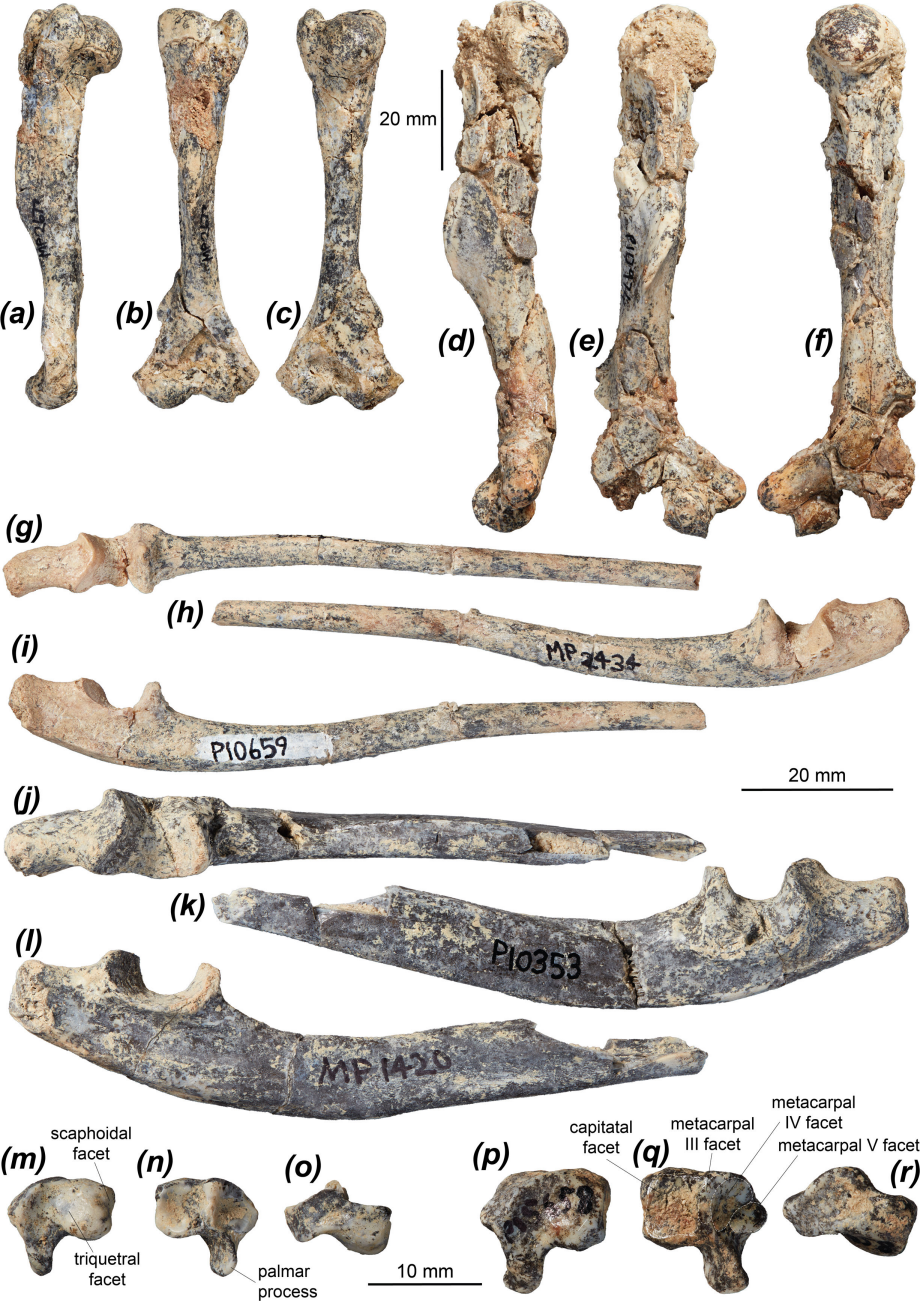
Forelimb elements of *Dorcopsoides fossilis* demonstrating size dimorphism in adult specimens. (*a*–*c*) Right humerus NTM P17479 in (*a*) lateral, (*b*) cranial and (*c*) caudal views; (*d*–*f*) partial right humerus NTM P10974 in (*d*) lateral, (*e*) cranial and (*f*) caudal views; (*g*–*i*) left ulna NTM P10659 in (*g*) cranial, (*h*) lateral and (*i*) medial views; (*j*–*l*) left ulna NTM P10353 in (*j*) cranial, (*k*) lateral and (*l*) medial views; (*m*–*o*) left hamatum NTM P13486 in (*m*) proximal, (*n*) distal and (*o*) cranial views; and (*p*–*r*) left hamatum NTM P15658 in (*p*) proximal, (*q*) distal and (*r*) cranial views.

Six semi-complete adult humeri (figures 1*d–g*, figure 2*a–f* and table 1) and an almost complete juvenile humerus (figure 1*f,**g*) are known, though the adult specimens are mostly crushed and warped. A female morphotype (figure 2*a*–*c*) and a male morphotype (figure 2*d*–*f*) are recognized, with the male morphotype larger, more robust and with better-developed muscle attachment sites. The humerus is moderately robust, straight in cranial view, with an elongate pectoral crest present on the cranial surface around the midpoint of the diaphysis. The humerus is more robust than that of *Ngamaroo archeri*, which has an elongate and distinctly straight humeral diaphysis, and more gracile than that of *L. fasciatus*. A small but distinct deltoid tubercle is present on the lateral surface midway between the proximal epiphysis and the start of the pectoral crest, better developed than in *Ng. archeri*, in which it is very low and slight. It also differs from the condition seen in *B. lesueur* and *Po. tridactylus*, which lack a distinct deltoid tubercle, instead possessing a deltopectoral crest that wraps from the lateral margin of the greater tubercle nearly to the proximal end of the medial supracondylar bridge.

**Table 1.** Dimensions in mm of partial humeri, ulnae and radii of *Dorcopsoides fossilis*.

specimen number	humerus		
	length	diaphyseal width at distal end of pectoral crest	distal width
NTM P6087	?	?	34.3
NTM P6090	?	9	?
NTM P6089	?	?	29.5
NTM P6091	?	12.9	?
NTM P5577	?	9.2	?
NTM P10974	106	10.2	?
NTM P17479	78.6	7	?

specimen number	ulna		
	olecranon length (from centre of trochlear notch)		height of ulna at centre of trochlear notch
NTM P6061	?		7.5
NTM P8711-3	15.4		8.3
NTM P6068	?		8.3
NTM P6060	18.5		10.7
NTM P6058	15.3		8
NTM P6065	15.6		7.8
NTM P6066	14.6		8.3
NTM P6059	?		7.8
NTM P6062	14.6		7.7
NTM P10353	18.5		11.5
NTM P10659	?		7.1

specimen number	radius		
	minimum diameter of proximal end		
NTM P879-3	9.7		
NTM P879-4	9.2		
NTM P6084	7.4		

The pectoral crest is narrow and raised, within a similar range to that of most compared macropodines. It is more raised and less laterally displaced than in *L. fasciatus*, *G. robustiter* and *Ng. archeri*. The distal end of the humerus is quite narrow and elongate, more so than in *S. brachyurus* and *De. lumholtzi*, but similar to the other compared macropodines and to *L. fasciatus.* The lateral supracondylar (epicondylar) crest (for the origin of the m. brachioradialis, which flexes the elbow, and mm. extensor carpi et digitorum, which extend the manus and digits) [85] is elongate and fairly narrow, with a slightly pointed proximal peak (figure 1*e*), similar to those of *No. eugenii* and *T. billardierii*. It is broader with a slightly more pointed proximal peak than in *M. fuliginosus*, *Pe. xanthopus, Du. vanheurni*, *Do. luctuosa*, *Do. muelleri*, *G. robustiter* and *Ng. archeri*, less proximally extensive than in *S. brachyurus*, *Do. muelleri*, *B. lesueur* and *Po. tridactylus*, narrower with a smaller proximal peak than in *S. brachyurus*, *L. fasciatus*, *B. lesueur* and *Po. tridactylus*, and narrower than in *De. lumholtzi*.

The olecranon fossa (figure 1*e*) is proximodistally short, broad and deep, abutting the caudal proximal margin of the capitulum and trochlea, which it is marginally narrower than. The olecranon fossa is similar to those of most macropodines and *L. fasciatus* but is deeper and broader relative to the width of the distal end than in *De. lumholtzi*, and is less proximally extensive than in *M. fuliginosus*, *No. eugenii* and *Pe. xanthopus*. The capitulum abuts the lateral epicondyle (figure 1*d*). The trochlea is separated from the medial epicondyle by a broad groove, as in all compared species except *T. billardierii*, *Do. luctuosa*, *Do. muelleri*, *Ng. archeri* and *B. lesueur*. The capitulum is strongly rounded and convex. The trochlea and capitulum are similar in combined width to those of most compared macropodines, but relatively narrower than in *T. billardierii*, which has the facets abutting both epicondyles. The capitulum is subequal in width to the trochlea; the capitulum is broader than the trochlea in *De. lumholtzi*, *Do. muelleri*, *Ha. puckridgi*, *G. robustiter*, *Ng. archeri* and the two compared potoroids. The trochlear concavity is variable in depth and shape and is not substantially different from that seen in any compared species.

#### 3.2.3. Radius

The radius of *Dd. fossilis* is known from five specimens, four of which preserve at most the proximal four-fifths of the element and one preserving a small segment of distal diaphysis and the distal epiphysis (figure 1*o*,*p* and table 1). The radial head (figure 1*k*) is circular, similar to all macropodines except the more oval radial head of *De. lumholtzi* and *Do. muelleri.* The humeral facet is gently concave, similar to all compared species except *De. lumholtzi* and *Po. tridactylus*, in which the humeral facet is more deeply concave and curves caudally at the sides. The radial tuberosity (figure 1*m*,*n*) is oval and prominent to very prominent, as in *No. eugenii*, *Du. vanheurni*, *Do. luctuosa* and *Do. muelleri*. In other macropodines and *Ha. puckridgi* the tuberosity is less prominent, and in *De. lumholtzi* it is more elongate. The radial tuberosity is more prominent, smaller in circumference relative to the width of the diaphysis, and situated closer to the radial head in *B. lesueur* and *Po. tridactylus*.

In the sole specimen preserving a substantial amount of the diaphysis distal to the radial tubercle (a juvenile, NTM P879-1, figure 1*h*–*j*) the diaphysis becomes increasingly craniocaudally compressed distally, similar to *T. billardierii* and *Du. vanheurni*, less so than in *S. brachyurus*, much less so than in *Po. tridactylus*, slightly more than in *De. lumholtzi*, and more so than in other compared macropodines. A low, narrow, elongate, distinct ridge is present on the craniomedial margin of the diaphysis, starting around one-quarter of the length of the diaphysis and extending at least to its midpoint, possibly for the insertion of the m. pronator teres, which pronates the manus [105]. This ridge is more prominent than in most compared macropodines, but similar to that of *M. fuliginosus*. In *S. brachyurus*, the ridge is similar in height but is shorter and curves laterally in its distal half. In *B. lesueur* and *Po. tridactylus*, this ridge is much more prominent, particularly in *Po. tridactylus*. On the caudolateral margin of the diaphysis, opposite the craniomedial ridge, is a thicker and more raised crest arising at a similar point and extending distally at least as far, possibly for the partial origin of the m. extensor carpi radialis, which extends metacarpals II and III [85]. This ridge is similar in *Du. vanheurni*, *Do. luctuosa* and *Do. muelleri*, shorter and less raised in *M. fuliginosus*, *No. eugenii*, *Pe. xanthopus* and *De. lumholtzi*, present as two narrower, parallel ridges in *T. billardierii*, and narrower, much more raised, and more elongate in *S. brachyurus* and the potoroids.

The distal epiphysis is broad and slightly craniocaudally compressed, similar to those of *Pe. xanthopus* and *Du. vanheurni*, with a gently concave and scaphoid (distal) facet that faces distally. It differs from those of *M. fuliginosus* and *T. billardierii* in having a less raised cranial tubercle, and from those of *De. lumholtzi* and *L. fasciatus* in having a less cranially tilted scaphoid facet. The styloid process is tall, narrow, and rounded in lateral view, similar to those of *T. billardierii* and *Du. vanheurni*. It differs from those *M. fuliginosus* and *No. eugenii* in being narrower, from *S. brachyurus*, *L. fasciatus* and *G. robustiter* in being slightly medially situated relative to the lateral margin of the distal epiphysis, and from *Pe. xanthopus* in being longer.

#### 3.2.4. Ulna

**Figure 3.**
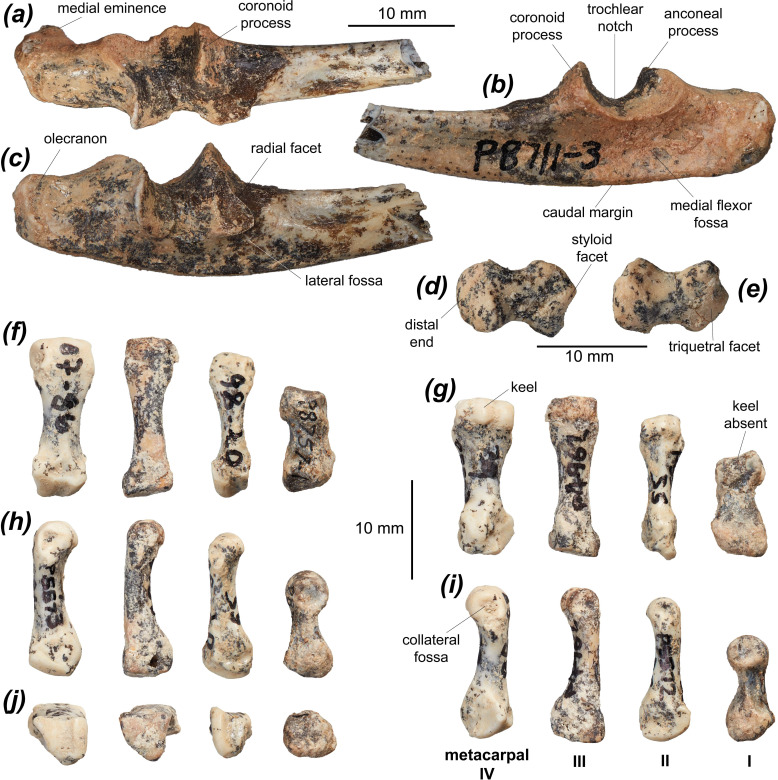
Forelimb elements of *Dorcopsoides fossilis*: (*a*–*c*) proximal right ulnar fragment NTM P8711-3 in (*a*) cranial, (*b*) medial and (*c*) lateral views; (*d*,*e*) right pisiform NTM P17668 in (*d*) dorsoproximal and (*e*) palmodistal views; and (*f*–*j*) left metacarpals IV NTM P4962, metacarpal III NTM P5573, metacarpal II NTM P5572 and metacarpal I NTM P8757-1 in (*f*) dorsal, (*g*) palmar, (*h*) medial, (*i*) lateral and (*j*) proximal views.

The ulna of *Dd. fossilis* is known from 12 partial specimens, preserving predominantly the proximal half of the element. A female morphotype (figure 2*g*–*i* and table 1) and a male morphotype (figure 2*j*–*l*) are recognized, with the male morphotype larger, considerably more robust and with better-developed muscle attachment sites. The olecranon (figure 3*c*) is elongate relative to the height of the proximal diaphysis, proportionally similar to those of *M. fuliginosus*, *No. eugenii*, *Pe. xanthopus*, *Du. vanheurni* and *L. fasciatus*, longer than in *De. lumholtzi*, *Do. luctuosa* and *Do. muelleri*, and shorter than in the two compared potoroids. The olecranon curves and flares distomedially with a medial eminence, similar to *No. eugenii*, *Du. vanheurni*, *Do. luctuosa*, *Do. muelleri* and *Po. tridactylus*, and slightly more than in *M. fuliginosus* and *L. fasciatus*. This eminence is likely associated with the insertion of the m. triceps brachii caput mediale or caput longum [85,105]. The trochlear notch is most similar to those of *No. eugenii* and *Do. luctuosa.* It is deeper than in *De. lumholtzi* and shallower than in *B. lesueur* and *Po. tridactylus*, lacking the dorsoproximal curvature of the peak of the coronoid process and the dorsodistal curvature of the anconeal process. *Hadronomas puckridgi* and *Dd. fossilis* have a similar trochlear notch, though the coronoid process (figure 2*b*) is taller in *Ha. puckridgi*. The morphology of the anconeal process in dorsal view is variable in *Dd. fossilis*, increasing in width and in mesial angle with age. The radial facet (figure 3) is roughly semicircular or crescentic and faces laterally in *Dd. fossilis* and most macropodines. In *S. brachyurus*, *Ng. archeri* and *Po. tridactylus* the radial notch is more rounded and dorsal facing, while in *L. fasciatus* it flares laterally at its caudodistal margin. The radial notch is less distally extensive than those of *De. lumholtzi* and *L. fasciatus*.

The caudal margin of the ulna is moderately convex (figure 3*b*), similar to that in *M. fuliginosus*, *No. eugenii* and *B. lesueur*, less convex than in *S. brachyurus*, *T. billardierii* and *De. lumholtzi*, and more so than in *Pe. xanthopus*, *Ng. archeri*, *Ha. puckridgi* and *Po. tridactylus*. The distal diaphysis curves slightly caudally, as in all compared species except *Po. tridactylus*, and more strongly than in *G. robustiter* and *Ng. archeri.* The proximal ulna beneath the trochlear notch is taller in *Ha. puckridgi* than in *Dd. fossilis*. The medial flexor fossa for the origin of the m. flexor digitorum profundus, which flexes the digits [85,105], extending distally and towards the olecranon from caudal to the trochlear notch on the medial surface, is elongate but fairly shallow, similar to those of *T. billardierii*, *Do. luctuosa* and *Do. muelleri*. In *M. fuliginosus*, *No. eugenii* and *Pe. xanthopus* this fossa is shorter and shallower, while it is deeper and more elongate in *S. brachyurus*, *De. lumholtzi*, *Du. vanheurni*, *L. fasciatus*, *G. robustiter*, *Ng. archeri* and the two compared potoroids, particularly along the olecranon.

#### 3.2.5. Pisiform

The pisiform of *Dd. fossilis* is known from a single specimen (figure 3*d,e*). The pisiform is short, robust and dorsopalmarly compressed with similarly bulbous proximal and distal ends and a strong waist between. It is most similar in its proportions to that of *Pe. xanthopus*, which differs in being slightly more elongate, and that of *L. fasciatus*, which differs in having a relatively slightly smaller proximal end. Those of *M. fuliginosus* and *No. eugenii* are more elongate with a relatively larger, broader distal end, those of *S. brachyurus* and *T. billardierii* have a stronger waist and a relatively much smaller proximal end, that of *De. lumholtzi* is more robust and has a weaker waist, that of *B. lesueur* is more elongate with a relatively smaller distal end, and that of *Po. tridactylus* is considerably more elongate with a relatively smaller proximal end.

#### 3.2.6. Hamatum

The hamatum of *Dd. fossilis* is known from three specimens. A female morphotype (figure 2*m*–*o*) and a male morphotype (figure 2*p*–*r*) are recognized, with the male morphotype being larger, more robust and with a better-developed palmar process. The hamatum is broad and fairly robust, most similar in its proportions to those of *T. billardierii* and *Pe. xanthopus*. Those of *S. brachyurus*, *B. lesueur* and *Po. tridactylus* are proximodistally shorter. The triquetral facet is broad and moderately concave, most similar to those of *M. fuliginosus* and *T. billardierii*. Those of *No. eugenii*, *S. brachyurus*, *De. lumholtzi* and *Po. tridactylus* are less concave, and those of *De. lumholtzi*, *B. lesueur* and *Po. tridactylus* are broader. The scaphoidal facet is narrow and strongly convex, continuous with the medial margin of the triquetral facet. This differs from those of *M. fuliginosus*, *No. eugenii*, *S. brachyurus*, *De. lumholtzi*, *T. billardierii*, *B. lesueur* and *Po. tridactylus*, which have a small but distinct margin dividing the two facets, and from *Pe. xanthopus*, which has the two facets distinctly separate. The capitatal facet is approximately square and moderately concave, very similar to most compared taxa. That of *De. lumholtzi* differs in being broader and more concave. The facets for metacarpals III, IV and V are semicontinuous. The metacarpal III facet is tall and very narrow, whereas the metacarpal IV facet is broader and more rounded and the metacarpal V facet is smaller and oval. These facets are very similar to those of *Pe. xanthopus. Setonix brachyurus*, *De. lumholtzi* and *Po. tridactylus* differ in having the metacarpal IV and V facets larger, relatively taller and more concave. *Macropus fuliginosus* differs in having a very small, dorsally restricted metacarpal III facet and a smaller, less distinct metacarpal V facet relative to the metacarpal IV facet. The palmar process is moderately tall (i.e. palmarly extensive), craniocaudally deep and transversely compressed, most similar to that of *T. billardierii*. Those of *M. fuliginosus*, *No. eugenii* and *De. lumholtzi* are shorter, while those of *S. brachyurus*, *B. lesueur* and *Po. tridactylus* are taller.

#### 3.2.7. Metacarpal I

Metacarpal I of *Dd. fossilis* is known from a single specimen (NTM P8757-1) (figure 3*f–i* and table 2). It is robust and strongly transversely asymmetrical with a slight waist, stronger on the lateral side of the diaphysis. It is similar in general morphology and proportions to those of *No. eugenii*, *S. brachyurus*, *Pe. xanthopus*, *Do. luctuosa* and *Do. muelleri*, is less robust with a stronger waist than that of *M. fuliginosus*, slightly less robust than those of *T. billardierii* and *L. fasciatus*, slightly more robust than in *De. lumholtzi*, and more robust than those of *B. lesueur* and *Po. tridactylus*. The proximal facet, which articulates with the trapezium, is slightly taller than it is broad and is marginally medially tilted, with its dorsal half convex and tilted moderately dorsally, similar to that of *B. lesueur*. In *M. fuliginosus* and *De. lumholtzi*, the trapezial facet is much broader and is gently concave, in *No. eugenii* it is narrower and gently concave, in *S. brachyurus* the facet curves above a shallow, mesial proximopalmar fossa, in *Pe. xanthopus*, *Do. luctuosa* and *Do. muelleri* it is slightly broader, and in *L. fasciatus* and *Po. tridactylus* it is narrower and approximately triangular. The mesial fossa is present but very slight and more palmarly situated in *Dd. fossilis*, *Do. luctuosa* and *Do. muelleri*. A dorsopalmarly tall, narrow (proximodistally short) and indistinct facet for metacarpal II abuts and is semicontinuous with the lateral margin of the trapezial facet, facing proximolaterally, similar to that of *No. eugenii*. This facet is less distinct and is continuous with the trapezial facet in *M. fuliginosus*, *Pe. xanthopus* and *De. lumholtzi*. It is less palmarly extensive in *S. brachyurus*, and larger and more distinct in *T. billardierii*, *Do. luctuosa*, *Do. muelleri*, *L. fasciatus*, *B. lesueur* and *Po. tridactylus.* There is a broad, raised mediopalmar tubercle on the proximal end, probably for ligamentous attachment. This tubercle is similar to those in *Do. luctuosa*, *Do. muelleri* and *L. fasciatus*, broader, less raised and more palmarly situated in those of *M. fuliginosus* and *No. eugenii*, smaller in those of *Pe. xanthopus*, *De. lumholtzi* and *B. lesueur*, and narrower in that of *Po. tridactylus*.

**Table 2.** Dimensions in mm of metacarpals I–IV of *Dorcopsoides fossilis*.

specimen number	element	length	distal width
NTM P8757-1	metacarpal I	11.8	5.5
NTM P5572	metacarpal II	16	4.9
NTM P8757-6	metacarpal II	17.8	6.8
NTM P5573-39	metacarpal III	17.7	6.9
NTM P5573-99	metacarpal III	16	5.9
NTM P6117	metacarpal III	?	7.8
NTM P8751-8	metacarpal III	17.1	6
NTM P8757-4	metacarpal III	15.4	5.2
NTM P5571-92	metacarpal III	16.1	5.3
NTM P5571-83	metacarpal III	16.6	6.2
NTM P4962	metacarpal IV	17.3	5.4

The distal end of metacarpal I is slightly narrower than the proximal end. The distal facet is slightly projected distally in its lateral section and is proximally extensive on both dorsal and palmar surfaces, extending to level with the proximal margin of the large, deep collateral fossae. The distal facet has a gently concave trochlea and lacks a keel, as in *T. billardierii*, *De. lumholtzi*, *Do. luctuosa*, *Do. muelleri* and *B. lesueur*. A rounded keel is present in *M. fuliginosus* and *No. eugenii*, and is present but very slight in *S. brachyurus*, *Pe. xanthopus* and *L. fasciatus*, while the distal facet in *Po. tridactylus* has no keel and is bulbously convex. The distal facet is otherwise similar in *No. eugenii*, *Do. luctuosa*, *Do. muelleri* and *B. lesueur*, less proximopalmarly extensive in *S. brachyurus* and *Po. tridactylus*, less proximally extensive in *T. billardierii* and *Pe. xanthopus*, and narrower and more palmarly concave in *De. lumholtzi*. The collateral fossae are shallower in *M. fuliginosus* and *No. eugenii*.

#### 3.2.8. Metacarpal II

Metacarpal II of *Dd. fossilis* is known from one adult specimen (NTM P5572) and a juvenile specimen lacking the distal epiphysis (NTM P5787-5) (figure 3*f–i* and table 2). It is narrow and elongate, with a long and distinct waist extending most of the length of the diaphysis. It is similar in proportion to those of *M. fuliginosus*, *No. eugenii*, *Pe. xanthopus*, *Do. luctuosa* and *Do. muelleri*, slightly more gracile than in *S. brachyurus* and *Du. vanheurni*, narrower and considerably more gracile than in *T. billardierii*, more robust than in *B. lesueur*, and much more robust than in *Po. tridactylus*. The waist is stronger than in *M. fuliginosus* and *De. lumholtzi.* The proximal end is tall, with the lateral half projected dorsally and proximolaterally by the tall facet for the capitatum (magnum), and transversely compressed, similar to those of *No. eugenii*, *Do. luctuosa* and *Do. muelleri*, though in the latter two the proximolateral projection is greater. The proximal end in *M. fuliginosus* and *De. lumholtzi* does not narrow palmarly, in *De. lumholtzi* is relatively narrower, and in *S. brachyurus* is broader due to a more medially projecting dorsomedial section on the proximal end. The facet for metacarpal I is tall and very narrow, medially facing, abutting the proximal margin on the medial surface and extending from the dorsal to the palmar margin. This facet is similar to those of *No. eugenii* and *Du. vanheurni*, broader but less distinct in *M. fuliginosus*, shorter in *S. brachyurus*, *Do. luctuosa* and *Do. muelleri*, relatively larger in *T. billardierii*, taller in *De. lumholtzi*, and relatively larger and broader in *B. lesueur* and *Po. tridactylus*. The facet for the trapezoid is tall, narrow and has an angular dorsopalmar groove deepening towards the dorsal margin. It extends onto the medial surface of the proximally projecting lateral process on the proximal end. This is similar in *No. eugenii*, *S. brachyurus* and *T. billardierii*, while in *M. fuliginosus* this facet is broader, in *Pe. xanthopus* the groove is deeper, in *De. lumholtzi* the groove is very slight and dorsally restricted, and in *B. lesueur* and *Po. tridactylus* the trapezoidal facet is small, rounded and restricted to the dorsal half of the proximal surface between the facets for metacarpal I and the capitatum. In *Do. luctuosa* and *Do. muelleri,* the trapezoidal facet is smaller and split into two separate facets—a larger, slightly convex and proximomedially facing lateral facet and a very small and narrow medial facet set either side of the dorsopalmar groove.

The facet for the capitatum is situated on the proximal surface of the proximolateral process, and is very tall, narrow and moderately convex, extending onto the dorsal surface of the proximal end. The facet is similar in *No. eugenii*, *S. brachyurus*, *Do. luctuosa* and *Do. muelleri*, while that of *M. fuliginosus* is less transversely convex and faces proximolaterally, those of *T. billardierii* and *Pe. xanthopus* are slightly broader and less transversely convex, that of *De. lumholtzi* is less convex and faces medially, that of *Du. vanheurni* is broader and less distinct, and those of *B. lesueur* and *Po. tridactylus* are continuous with the metacarpal III facet. In *Po. tridactylus* the capitatal facet is small, rounded and palmolaterally situated. The facet for metacarpal III is on the dorsal half of the lateral surface of the proximolateral process, and is fairly small, indistinct and rounded. This facet is similar to those of *No. eugenii*, *T. billardierii* and *Pe. xanthopus* and is larger and more palmarly extensive in *M. fuliginosus*, taller in *De. lumholtzi*, and broader and relatively larger in *S. brachyurus*, *Du. vanheurni*, *Do. luctuosa*, *Do. muelleri*, *B. lesueur* and *Po. tridactylus*.

The distal end is slightly broader than the proximal end. The distal facet has a prominent, rounded keel and is proximally extensive on both dorsal and palmar surfaces, extending to level with the proximal margins of the large, shallow collateral fossae. The collateral fossae are similar to those of *S. brachyurus*, *T. billardierii*, *Du. vanheurni*, *Do. luctuosa* and *Do. muelleri*, much shallower in *M. fuliginosus* and *Po. tridactylus*, slightly shallower in *Pe. xanthopus*, and deeper in *De. lumholtzi* and *B. lesueur.* The facet and keel are similarly developed in *Du. vanheurni*, while in *M. fuliginosus*, *No. eugenii*, *Do. luctuosa* and *Do. muelleri* the facet is less proximally extensive, in *M. fuliginosus* and *Po. tridactylus* the keel is more prominent, in *S. brachyurus*, *T. billardierii* and *Pe. xanthopus* the facet is less proximopalmarly extensive, in *T. billardierii* the keel is less prominent, in *De. lumholtzi* the facet is more convex and the keel is proximodistally shorter and more rounded, and in *B. lesueur* and *Po. tridactylus* the facet is more proximodorsally extensive and the keel is narrower.

#### 3.2.9. Metacarpal III

Metacarpal III of *Dd. fossilis* is known from a single adult specimen (NTM P4962) with a cracked proximopalmar section (figure 3*f–i* and table 2). It is narrow and elongate, with gentle narrowing to a slight waist just distal of the midpoint of the diaphysis. It is subequal in length to the metacarpal IV specimens but is more gracile. The degree of gracility is similar in *M. fuliginosus*, *No. eugenii*, *Pe. xanthopus*, *De. lumholtzi*, *L. fasciatus* and *B. lesueur*, while in *S. brachyurus*, *T. billardierii*, *Do. luctuosa* and *Do. muelleri* this metacarpal is more robust. The proximal end is slightly taller than is it wide, approximately triangular in proximal view. The proximal end has a swollen, projected palmar tubercle and a slightly laterally projected lateral side amounting to a gently raised tubercle, housing the metacarpal IV facet, most similar to *Pe. xanthopus*. In *M. fuliginosus*, *No. eugenii*, *Do. luctuosa* and *Do. muelleri* this lateral tubercle is much larger and more proximolaterally projected, forming a distinct proximolateral process, in *De. lumholtzi* the lateral tubercle is smaller, and in *Do. luctuosa*, *Do. muelleri*, *B. lesueur* and *Po. tridactylus* the proximal end is substantially broader relative to its height. There is a small, indistinct, rounded facet for metacarpal II situated on the medial surface abutting the proximodorsal corner. This facet is similar in *S. brachyurus* and *T. billardierii*, larger and more distinct in *M. fuliginosus*, *Do. luctuosa*, *Do. muelleri*, *B. lesueur* and *Po. tridactylus*, and slightly taller and narrower in *No. eugenii*, *Pe. xanthopus* and *De. lumholtzi*.

The capitatal facet is large and approximately triangular with a level dorsal margin, occupying the entire proximal surface of the metacarpal. It has a gentle dorsomesial concavity, being otherwise flat and facing proximally and slightly medially. The capitatal facet is more deeply dorsomesially concave in *M. fuliginosus, S. brachyurus*, *Pe. xanthopus*, *De. lumholtzi* and *Po. tridactylus*, more convex in *No. eugenii*, quadrilateral in *T. billardierii* though with a narrow palmar section, narrower in *De. lumholtzi*, and broader and more rounded palmarly in *L. fasciatus*. In *Do. luctuosa* and *Do. muelleri* this facet is more convex on its lateral half and has a strongly convex dorsal margin either side of the mesial concavity. In *B. lesueur* the capitatal facet is much broader and has a small, deep, palmarly situated fossa situated mesially in the palmar section. A tall and very narrow facet for the hamatum occupies the lateral margin of the proximal end, abutting the lateral margin of the capitatal facet. This facet is similar in *Pe. xanthopus*, broader and less tall in *M. fuliginosus*, *Do. luctuosa* and *Do. muelleri* in which it occupies the proximolateral surface of the large proximolateral process, smaller, dorsally restricted and proximolaterally facing in *Po. tridactylus*, and absent in *De. lumholtzi* and *B. lesueur*. Immediately distal to the lateral margin of the hamatal facet is a large, shallow fossa opening distolaterally, housing the tall, dorsally broad and palmarly narrow facet for metacarpal IV, which faces distally and slightly laterally. The metacarpal IV facet is similar in *S. brachyurus*, *T. billardierii*, *Pe. xanthopus*, *De. lumholtzi*, *L. fasciatus*, *B. lesueur* and *Po. tridactylus* but faces more laterally, in *M. fuliginosus*, *No. eugenii*, *Do. luctuosa* and *Do. muelleri* is larger, more distinct and more distal facing, and in *S. brachyurus* is broader and more dorsally restricted.

The diaphysis broadens smoothly to a distal end subequal in width to the proximal end. *Dorcopsoides fossilis* lacks the small, pointed eminence present in *L. fasciatus*, *B. lesueur* and *Po. tridactylus* midway along the diaphysis on the dorsomedial surface, pointing medially. The distal facet is proximally extensive on both dorsal and palmar surfaces, extending to level with the proximal margins of the large, shallow collateral fossae, and has a prominent, rounded keel. In *De. lumholtzi*, *L. fasciatus* and *B. lesueur*, the collateral fossae are larger and deeper. The facet and keel are similarly developed in *Du. vanheurni* and *B. lesueur*, while in *M. fuliginosus*, *No. eugenii*, *Pe. xanthopus* and *De. lumholtzi* the facet is less proximally extensive, in *M. fuliginosus*, *No. eugenii*, *Do. luctuosa*, *Do. muelleri* and *Po. tridactylus* the keel is more prominent, in *S. brachyurus* the facet is narrower relative to the width of the distal end and is less proximopalmarly extensive with the keel more prominent and rounded, in *T. billardierii* the keel is less prominent, in *Pe. xanthopus* the keel is broader, in *De. lumholtzi* the distal surface of the facet is tilted medially, and in *Po. tridactylus* the facet is less proximally extensive.

#### 3.2.10. Metacarpal IV

Metacarpal IV of *Dd. fossilis* is known from six specimens, including one juvenile. The fourth metacarpal is large and fairly robust (figure 3*f–i* and table 2). It is subequal in length to metacarpal III but notably more robust and narrows to a gentle waist around midway along the diaphysis before broadening markedly to the distal end. Metacarpal IV is similarly robust in *M. fuliginosus*, *No. eugenii*, *S. brachyurus*, *T. billardierii*, *Pe. xanthopus*, *L. fasciatus*, *B. lesueur* and *Po. tridactylus* and is more gracile in *De. lumholtzi.* The proximal end is slightly taller than it is wide, approaching triangular in proximal view, with a laterally flared dorsal component, a level dorsal margin and a sub-dorsopalmarly aligned lateral margin, and with a swollen and projected palmar tubercle. This is similar in *T. billardierii*, *Pe. xanthopus* and *L. fasciatus.* The palmar tubercle is smaller and less distally extensive in *M. fuliginosus*, *S. brachyurus*, *B. lesueur* and *Po. tridactylus*, narrower in *No. eugenii*, and slightly broader and more palmarly projected in *De. lumholtzi.* The dorsolateral margin of the proximal end is more laterally prominent in *M. fuliginosus*, *Pe. xanthopus* and *B. lesueur*, and less laterally prominent in *De. lumholtzi.* The facet for metacarpal III is tall and transversely convex, wrapping around the proximolateral corner of the proximal end and separated from the hamatal facet by a narrow, shallow groove, similar to *S. brachyurus* and *Pe. xanthopus*, though in the latter the groove is broader. In *M. fuliginosus* this facet is flat, broader, more distinct and faces proximally. In *No. eugenii* it is similar to in *Dd. fossilis* but is at right angles rather than convex, in *T. billardierii*, *De. lumholtzi* and *Po. tridactylus* it is flat and restricted almost entirely to the lateral surface, in *Po. tridactylus* it is larger and more rounded, and in *L. fasciatus* and *B. lesueur* the facets are separated by a deep but dorsally restricted groove.

The hamatal facet is large, faces proximally, and varies from triangular to quadrilateral with a narrowed palmar section, similar to in *No. eugenii*, *Pe. xanthopus* and *L. fasciatus* but slightly less dorsopalmarly convex. In *M. fuliginosus*, *S. brachyurus* and *B. lesueur* this facet is broader and gently convex in its dorsal component, in *T. billardierii* it is gently laterally tilted, in *De. lumholtzi* it occupies the whole proximal surface with a concave, mesially situated dorsopalmar groove, and in *Po. tridactylus* it occupies almost all of the proximal surface, with the larger medial section deeply, angularly concave and the smaller lateral section gently convex. The facet for metacarpal V is oblong to lenticular, gently concave, facing laterally and slightly palmarly on the lateral surface of the proximal end and extending dorsodistally from the proximal margin, situated in a large, shallow fossa opening laterally and slightly distally, similar to that of *L. fasciatus*. In *M. fuliginosus* the metacarpal V facet is rounder, less distally extensive, and faces more distally and palmarly due to its placement on the proximal dorsolateral prominence, in *No. eugenii* it is taller and less distally extensive, in *S. brachyurus* it is less dorsodistally extensive, in *T. billardierii* it is more distal facing, in *Pe. xanthopus* it is slightly more palmar facing, in *De. lumholtzi* is more lateral facing, and in *B. lesueur* it is more concave. In *Po. tridactylus* the metacarpal V facet is similarly sized and tilted to that of *Dd. fossilis*, but also has a secondary metacarpal V facet on the proximolateral base of the diaphysis, similar in size to the typical metacarpal V facet and approximately triangular.

The distal end is notably broader than the proximal end, unlike in *M. fuliginosus*, *Pe. xanthopus* and *B. lesueur* in which the two ends are subequal in width. The distal facet is proximally extensive on both dorsal and palmar surfaces, extending almost to level with the proximal margin of the large, shallow collateral fossae (figure 2*j*), is gently distally convex, and has a prominent, rounded keel (figure 2*h*), most similar to those of *No. eugenii* and *Pe. xanthopus*. In *M. fuliginosus* the lateral third of the distal facet is smaller and the collateral fossae are almost absent, in *S. brachyurus* the distal facet is distally convex and less proximopalmarly extensive with the keel more prominent and rounded, in *T. billardierii* the collateral fossae are smaller and deeper, in *De. lumholtzi* the facet is slightly more distally convex and the collateral fossae are larger and deeper, in *L. fasciatus* the keel is narrower and less prominent and the collateral fossae are larger and deeper, in *B. lesueur* the collateral fossae are deeper and the keel is less palmarly prominent than the parallel medial and lateral palmar prominences, which also protrude slightly distally such that the distal facet is very slightly concave, and in *Po. tridactylus* the facet is less proximally extensive and the keel is narrower and slightly distally prominent.

#### 3.2.11. Pelvis

**Figure 4.**
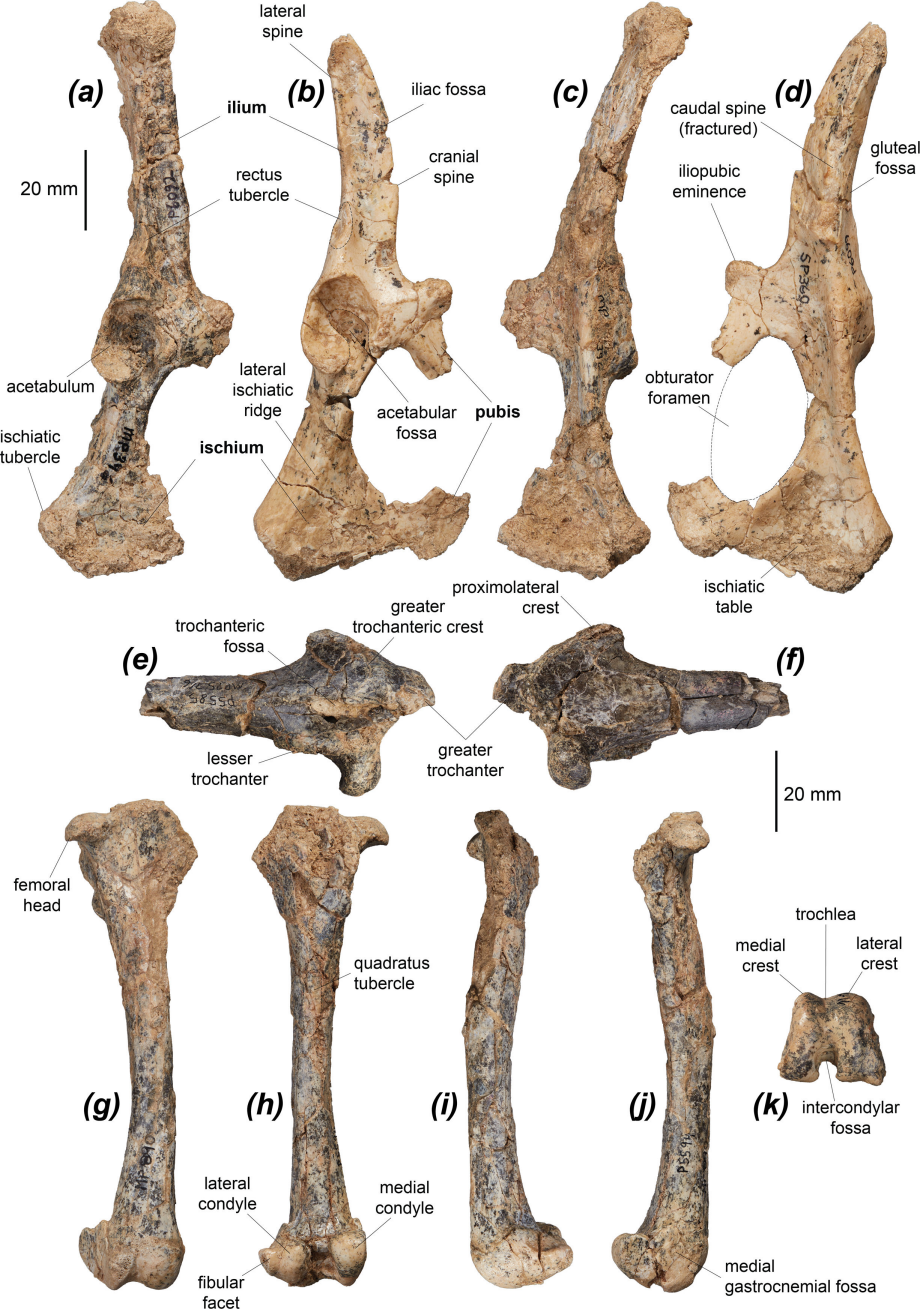
Proximal hindlimb elements of *Dorcopsoides fossilis*: (*a*–*d*) partial right pelves (*a*,*c*) NTM P6092 and (*b*,*d*) NTM P6099 in (*a*,*b*) lateral and (*c*,*d*) dorsomedial views; (*e*,*f*) proximal left femoral fragment NTM P5585 in (*e*) caudal and (*f*) cranial views; and (*g*–*k*) left femur NTM P5594 in (*g*) cranial, (*h*) caudal, (*i*) lateral, (*j*) medial and (*k*) distal views. Bold labels on pelvis indicate elements.

The length of the ilium relative to the ischium in *Dd. fossilis* is most similar to those of *Pe. xanthopus*, *T. billardierii*, *Do. luctuosa* and *Do. muelleri*, which have a relatively longer ilium than in *M. fuliginosus*, *No. eugenii* and *L. fasciatus*, and relatively shorter ilium than in *De. lumholtzi* and the two potoroids. *Dendrolagus lumholtzi* and *Po. tridactylus* have a relatively longer ischium again (figure 4 and table 3). The ilium curves very gently caudodorsally, similar to all compared macropodoids except *M. fuliginosus* and *No. eugenii*, which have a more caudodorsally deflected ilium. The caudal iliac spine is not preserved in any specimen, so the depth of the gluteal fossa cannot be gauged. The cranial iliac spine is deep (figure 4*b*). The iliac fossa, located between the lateral and the cranial iliac spines and for the origin of the m. iliacus, a major hip flexor [87], is more deeply concave than in *S. brachyurus*, *De. lumholtzi* and the two potoroids, more similar to those of the other compared species. The large rectus tubercle for the origin of the m. rectus femoris, which acts to flex the hip [87,106], is distinctly separate from the rim of the acetabulum. This is unlike in *S. brachyurus*, *De. lumholtzi*, *Du. vanheurni*, *Do. luctuosa*, *Do. muelleri*, *B. lesueur* and *Po. tridactylus*, in which it abuts the acetabular rim or is immediately adjacent to it.

**Table 3.** Dimensions in mm of pelves, femora and tibiae of *Dorcopsoides fossilis.*

specimen number	pelvis				
	ilium length (centre of acetabulum to lateral tip of iliac crest, excluding epiphyses)		ischium length (centre of acetabulum to ischiatic tuberosity, excluding epiphyses)		
NTM P6092	87.4		62.8		
NTM P6099	?		66.6		

specimen number	femur				
	total articular length	minimum diaphysis width	distal epiphysis width across caudal margin		length to width index
NTM P18448	162.1	14.8	35.5		11
NTM P5594	152.0	13.1	34.8		11.6
NTM P5595	146.0	13.4	32.3		10.9
NTM P18032	?	?	27.6		?

specimen number	tibia				
	length (excluding epiphyses)	minimum diaphysis width	width across proximal epiphysis	width across proximal base of distal epiphysis	length of cnemial crest (excluding proximal epiphysis)
NTM P4922	?	?	?	19.2	?
NTM P5587-1	?	?	?	17.7	?
NTM P5587-2	?	?	?	18	?
NTM P5569	?	?	29.2	?	?
NTM P5576	?	?	29.3	?	?
NTM P4369	?	?	?	18.2	?
NTM P6114	?	?	28.9	?	?
NTM P92137	?	15.9	?	?	~60
NTM P4322	?	14.3	?	?	?
NTM P18032	160	8.6	?	?	?

The acetabulum is oval and craniocaudally elongate (figure 4*a*), differing most from the more rounded acetabula of *De. lumholtzi* and *Ha. puckridgi*. The ischiatic portion of the acetabulum is larger than the pubic portion as in the other compared macropodines and in *L. fasciatus*. In *Ha. puckridgi*, *B. lesueur* and *Po. tridactylus* the two portions are more similar in size. The acetabular foramen is much smaller than that of *Ha. puckridgi.* The iliopubic (pectineal) eminence, from which the m. pectineus (which aids in hip adduction and lateral femoral rotation) originates [87], is well developed, prominent and rounded-square in shape (figure 4*d*). This is similar to all compared species except *S. brachyurus* and *De. lumholtzi*, in which this eminence is less prominent.

The obturator foramen (figure 4*d*) is more rounded (i.e. less elongated oval) than in *De. lumholtzi*, *Do. luctuosa* and the potoroids. The proximal ischium below the acetabulum is fairly deep and robust, more so than in *No. eugenii*, *Pe. xanthopus*, *T. billardierii* and the two potoroids. In this respect, it is similar to *M. fuliginosus*, *S. brachyurus* and *De. lumholtzi*, from which it differs in having a low, angular ridge extending along the lateral surface of the ischium from caudoventral to the acetabular fossa to caudoventral to the ischiatic tubercle, parallel with the caudodorsal margin of the ischium. The section of the ischium caudal to this ridge is deeper than in compared non-dorcopsins. In *Dd. fossilis*, *No. eugenii* and *L. fasciatus* the ischium forms a rounded point on its caudoventral margin, medial to the ischiatic tubercle, rather than being gently, smoothly convex as in all other compared macropodines, and strongly convex in the compared potoroids.

#### 3.2.12. Femur

Many partial femora of *Dd. fossilis* are known, but none preserve both epiphyses and all bar two distal epiphyses are crushed and warped to some degree (figure 4*e–k* and table 3). The proximal end of the femur is broad relative to its length, due to the medial projection of a large lesser trochanter and the lateral projection of a broad, well-developed greater trochanteric crest (figure 4*e*). The greater trochanter is broad, with a small cranial eminence, similar to those of other compared species except *M. fuliginosus*, which lacks this eminence. The greater trochanteric crest is broad, broadening distally, such that it is around twice its proximal width, to end at a thick, laterally prominent proximolateral crest (figure 4*f*). The m. gluteus superficialis, a hip joint extensor and abductor, inserts onto the proximal greater trochanteric crest, while the enlarged proximolateral crest is probably for insertion of the cranial head of the m. gluteus medius (a hip extensor) on the cranial surface and for the origin of the m. vastus lateralis, a knee extensor, on its caudal surface [86,87,106]. The proximolateral crest is most similar to those of *Du. vanheurni*, *Do. luctuosa* and *Do. muelleri*, but is less distally extensive, slightly broader and more prominent in *Dd. fossilis*. The crest is weakly developed and narrows distally in *M. fuliginosus*, *No. eugenii* and *L. fasciatus. Setonix brachyurus* and *Ngamaroo archeri* have slightly less broad and protrusive proximolateral crests, more distally extensive than in *Dd. fossilis*. It is considerably broader and more distally extensive than in *De. lumholtzi*, and broader than in the compared potoroids. The femoral head is rounded, and smaller relative to the size of the proximal end of the femur, unlike *De. lumholtzi* and *Ng. archeri*, which have proportionally much larger femoral heads. *Dendrolagus lumholtzi* also has a proportionally shorter greater trochanter. The femoral head is projected and rotated very slightly cranially, similar to all compared species. The lesser trochanter, to which the hip flexor m. iliopsoas (the combined m. iliacus and m. psoas major) inserts, is medially prominent and angled caudomedially. This is unlike the more strongly caudally angled lesser trochanter in *B. lesueur* and *Po. tridactylus*. The lesser trochanter is less medially prominent in all other compared species except *Du. vanheurni*, *Do. luctuosa*, *Do. muelleri* and *Ng. archeri* and is less distally extensive relative to the femoral head than in *S. brachyurus*, *De. lumholtzi*, *B. lesueur* and *Po. tridactylus*.

The femur is gracile, most similar in robustness to *S. brachyurus* and *Ng. archeri*. The femora of *M. fuliginosus* and *No. eugenii* are more robust, while those of *Pe. xanthopus*, *T. billardierii*, *De. lumholtzi* and the compared potoroids are more gracile. The quadratus tubercle, for the insertion of the m. quadratus femoris, which extends the hip joint [87,106], is positioned proximally on the caudal surface of the diaphysis, around one-third of the distance along the diaphysis from the proximal end (figure 4*h*). This is similar to the position of the tubercle in *M. fuliginosus*, *No. eugenii*, *T. billardierii*, *Pe. xanthopus* and *B. lesueur*, and more proximally situated than in *S. brachyurus*, *Do. luctuosa*, *L. fasciatus*, *Ng. archeri* and *Po. tridactylus*. In *De. lumholtzi* the quadratus tubercle is larger and positioned more distally and more medially, and in the potoroids the tubercle is smaller and much more raised.

The distal epiphysis is similar in height to its width, as in all compared species except *M. fuliginosus*, in which it is relatively narrower, and *De. lumholtzi*, which has a relatively broader distal epiphysis. The distal epiphysis projects more distally and less caudally in lateral view than in *Po. tridactylus* and to a lesser degree in *S. brachyurus* and *De. lumholtzi*. The lateral trochlear crest is broad and rounded, subequal in height to the medial trochlear crest (figure 4*k*). The medial trochlear crest is narrower and more angular, similar to those of *M. fuliginosus* and *No. eugenii*, and broader than in *S. brachyurus*, *T. billardierii*, *Pe. xanthopus*, *De. lumholtzi*, *B. lesueur* and *Po. tridactylus*. The medial crest and medial condyle are smoothly rounded across their distal surface, lacking the ridge running craniocaudally down the distomedial margin that is present in *S. brachyurus*, *Pe. xanthopus*, *De. lumholtzi*, *T. billardierii*, *Du. vanheurni*, *L. fasciatus*, *G. robustiter* and the two potoroids. The trochlea is moderately broad and skewed slightly medially (figure 4*k*), similar to *M. fuliginosus* and *No. eugenii*, and less medially skewed than in the other compared taxa.

#### 3.2.13. Tibia

**Figure 5.**
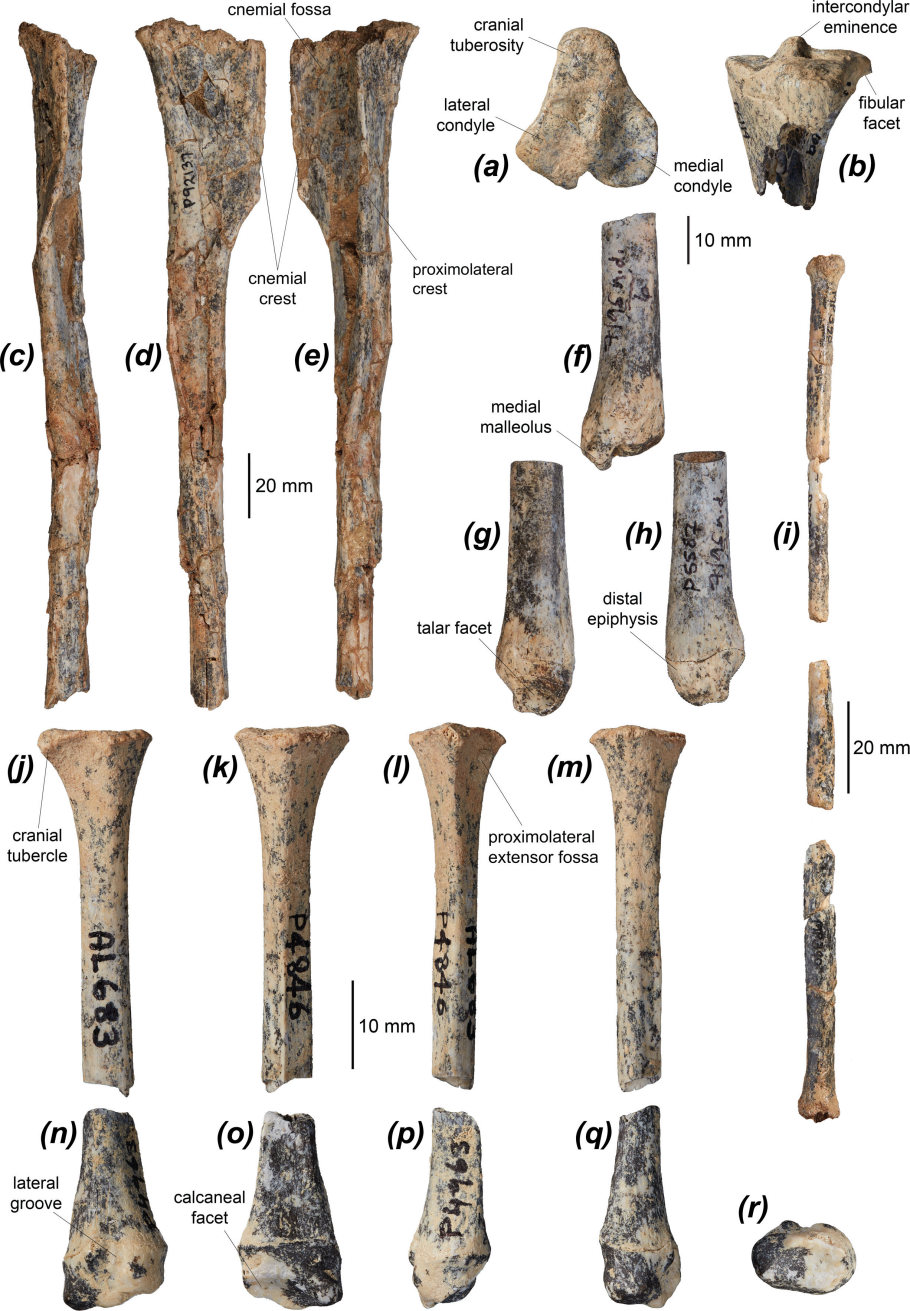
Distal hindlimb elements of *Dorcopsoides fossilis*: (*a*,*b*) proximal left tibial fragment in (*a*) proximal and (*b*) cranial views; (*c*–*e*) left tibia NTM P92137 (missing epiphyses) in (*c*) cranial, (*d*) medial and (*e*) lateral views; (*f*–*h*) distal left tibial fragment NTM P5587 in (*f*) cranial, (*g*) lateral and (*h*) medial views; (*i*) partial left fibula in lateral view; (*j*–*m*) proximal left fibular fragment NTM P4846 (missing proximal epiphysis) in (*j*) lateral, (*k*) medial, (*l*) cranial and (*m*) caudal views; and (*n*–*r*) distal left fibular fragment NTM P4963 in (*n*) lateral, (*o*) medial, (*p*) caudal, (*q*) cranial and (*r*) distal views.

**Figure 6.**
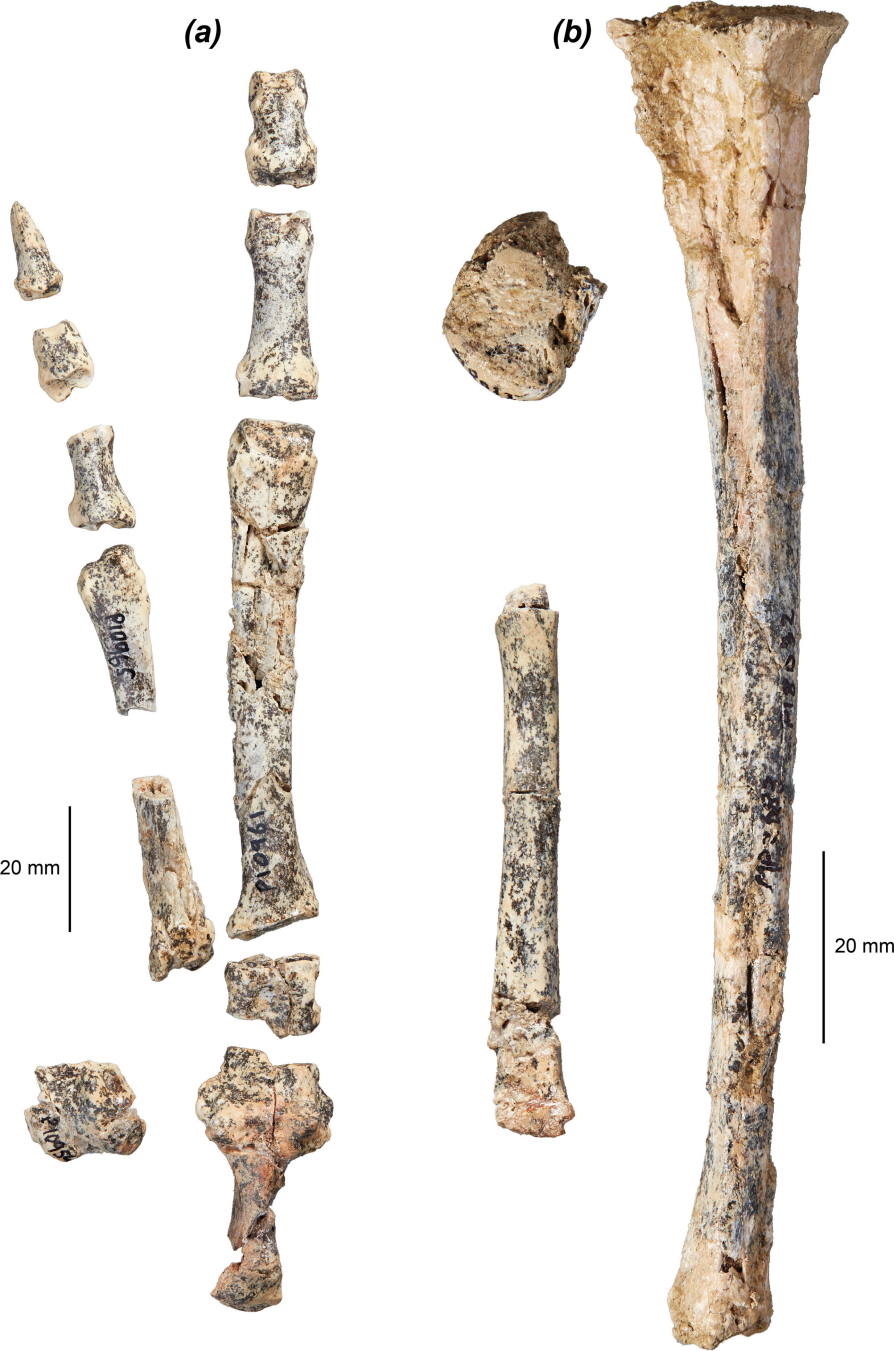
Associated hindlimb specimens of *Dorcopsoides fossilis*: (*a*) near-complete left pes NTM P10954–P10956 and NTM P10961–P10968 in dorsal view, showing partial calcaneus, talus and cuboid, metatarsal IV, proximal and middle fourth phalanges, proximal and distal segments of metatarsal V, partial proximal fifth phalanx, and middle and distal fifth phalanges; and (*b*) partial juvenile left hindlimb NTM P18032 in lateral view, preserving distal femoral epiphysis, tibia missing proximal epiphysis, and metatarsal IV missing distal epiphysis.

The tibia of *Dd. fossilis* is elongate, though as it is incompletely known its relative robustness is unknown (figure 5*a–h* and table 3). A partial associated juvenile specimen is known: NTM P18032, which includes a distal femoral epiphysis, a tibia missing its proximal epiphysis and a partial metatarsal IV (figure 6*b*). The proximal epiphysis of the tibia (figure 5*a*,*b*) is deeper than its width, subtriangular in shape, and has a rounded cranial tuberosity, most similar to *No. eugenii*, *T. billardierii*, *Pe. xanthopus* and *L. fasciatus*. That of *M. fuliginosus* is relatively narrower due to its narrow, cranially projected cranial tuberosity. *Dorcopsis luctuosa* and *Do. muelleri* have broader proximal epiphyses relative to their length, while that of *Du. vanheurni* is shallower again, similar to those of *S. brachyurus*, *De. lumholtzi* and the two potoroids. The intercondylar eminence (figure 5*b*) is tall and narrow, but comparatively shorter than in *M. fuliginosus* and *No. eugenii*. The medial condyle is reniform and moderately concave on its proximal surface, similar to all compared species except *S. brachyurus*, which lacks this concavity. The proximal articular surface is tilted caudally, but to a lesser extent than in *S. brachyurus*, *L. fasciatus*, *B. lesueur* and *Po. tridactylus*, more similar to those of *M. fuliginosus*, *No. eugenii*, *T. billardierii*, *Pe. xanthopus* and *Ha. puckridgi*. That of *De. lumholtzi* is slightly caudally tilted but also convex in lateral view.

Two tibiae of *Dd. fossilis* preserve the proximal ~four-fifths of the diaphysis (NTM P92137 and NTM P4322). There is a large, elongate cnemial crest that decreases very slightly in depth towards a distinct distal peak, then rapidly merges into the diaphysis (figure 5*d*,*e*). The cnemial crest, the lateral surface of which (cnemial fossa) is for the origin of the m. tibialis cranialis (which dorsiflexes and inverts the pes [87]) is one-quarter to one-fifth of the length of the tibia, similar to in *S. brachyurus*, *T. billardierii*, *Du. vanheurni*, *Do. luctuosa*, relatively slightly longer than in *M. fuliginosus*, *No. eugenii*, *Pe. xanthopus* and *L. fasciatus*, and relatively shorter than in *Do. muelleri*, *Ha. puckridgi* and *Ng. archeri*. The cnemial crest has a distinct distal peak, more distinct than in *S. brachyurus*, *T. billardierii*, *De. lumholtzi*, *Ha. puckridgi* and *Ng. archeri*. That of *Du. vanheurni* merges more gradually into the diaphysis distally. Those of *M. fuliginosus*, *No. eugenii*, *T. billardierii* and *Pe. xanthopus* become shallower distally, and that of *B. lesueur* is shallower. The proximolateral crest, which cranially borders the elongate fossa for the insertion of the knee-flexor m. popliteus [87], is fairly thin (i.e. craniocaudally shallow) and raised. This is similar to those of all compared species except *S. brachyurus*, *De. lumholtzi*, *L. fasciatus*, *Ng. archeri* and *B. lesueur*, in which it is less raised. Those of *Du. vanheurni* and *Po. tridactylus* are marginally thicker. The relative length of the distal fibular facet of *Dd. fossilis* is not known. The diaphysis expands distally, and is roughly circular in cross-section. The diaphysis is slightly bowed in known specimens, as in *S. brachyurus*, *De. lumholtzi* and the potoroids, but this may be taphonomically affected. The talar facet (figure 5*g*) is roughly rectangular, relatively narrower than in *M. fuliginosus*. The depth of the two talar grooves mirrors the relative heights of the medial and lateral crests on the talus. A rounded caudal expansion near the medial malleolus is present on the talar facet, as in those of *No. eugenii*, *Pe. xanthopus* and *De. lumholtzi*, and to a lesser extent in *S. brachyurus* and *Do. luctuosa*. The medial malleolus does not flare medially as much as in *S. brachyurus*, *L. fasciatus* and the two potoroids.

#### 3.2.14. Fibula

The proximal end of the fibula has a prominent craniomedial tuberosity (figure 5*j*) that is similar in most compared taxa but reduced in *S. brachyurus*, *B. lesueur* and *Po. tridactylus*. The fibular diaphysis is medially concave for tibial articulation distally as in all compared species except *Po. tridactylus*, and is concave proximolaterally likely for the partial origin of m. extensor digitorum IV [87]. *Dorcopsoides fossilis* has a narrow proximodistally aligned groove on the lateral surface of the distal epiphysis (figure 5*n*), probably a pathway for the m. fibularis (peroneus) longus et brevis (which dorsiflexes the pes) and/or the m. extensor digitorum lateralis (which extends and abducts digit V) [86,87]. A small, rounded tubercle is present on the epiphysis on the cranial and caudal sides of this groove, for the attachments of the peroneal tendon of the fibula [82]. These tubercles contribute to the depth of the groove. This distolateral groove and the two tubercles are similarly developed in all compared species except *M. fuliginosus*, *No. eugenii* and *Pe. xanthopus*, in which the groove is shallower and narrower and the peroneal tubercles slightly smaller, in *L. fasciatus* and *Ng. archeri*, in which it is longer, broader and deeper, and in *Po. tridactylus*, in which it is very slight.

#### 3.2.15. Talus (astragalus)

**Figure 7.**
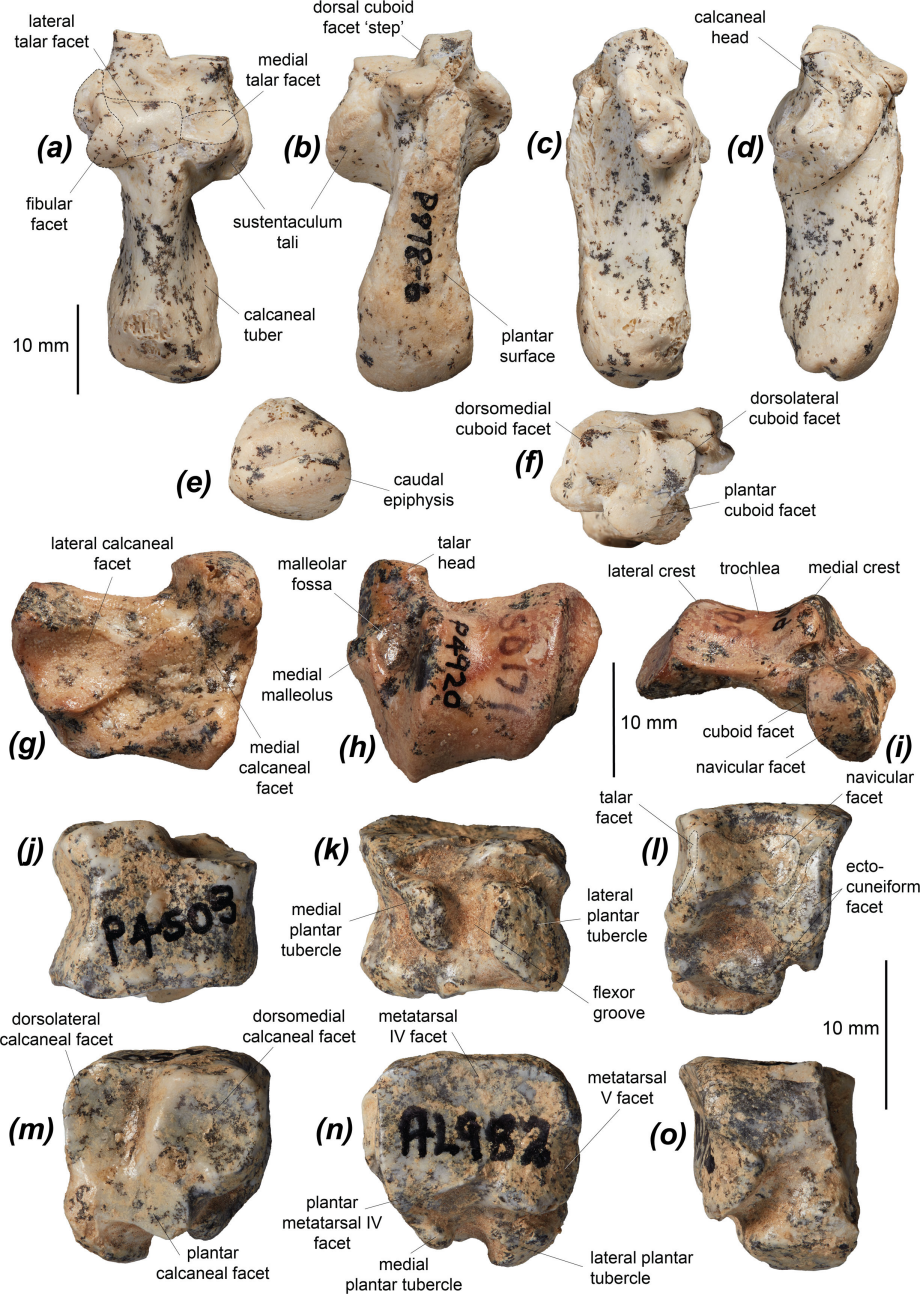
Pedal elements of *Dorcopsoides fossilis*: (*a*–*f*) left calcaneus NTM P878-6 in (*a*) dorsal, (*b*) plantar, (*c*) lateral, (*d*) medial, (*e*) caudal and (*f*) cranial views; (*g*–*i*) right talus (astragalus) NTM P4920 in (*g*) plantar, (*h*) dorsal and (*i*) cranial views; and (*j*–*o*) left cuboid NTM P4503 in (*j*) dorsal, (*k*) plantar, (*l*) medial, (*m*) proximal, (*n*) distal and (*o*) lateral views.

The talus of *Dd. fossilis* is broader than it is deep (figure 7 and table 4). The proportions of the talus in dorsal view are similar to those of *No. eugenii* and *T. billardierii*, craniocaudally shorter than those of *M. fuliginosus* and *Pe. xanthopus*, slightly longer than those of *Do. luctuosa* and *Do. muelleri*, and longer than those of *De. lumholtzi*, *L. fasciatus*, *B. lesueur* and *Po. tridactylus*. The trochlear crests are orientated slightly obliquely relative to the sagittal plane (figure 7*h*), most like *M. fuliginosus* and *No. eugenii*; not as strongly oblique as in *De. lumholtzi*, *S. brachyurus*, *Do. luctuosa*, *Do. muelleri*, *B. lesueur* or *Po. tridactylus*, nor craniocaudally aligned as in *Ha. puckridgi*. The medial trochlear crest is slightly taller than the lateral crest (figure 7*i*), as in all compared macropodines except *De. lumholtzi*, which has subequal trochlear crests. The trochlea is moderately deep with a slightly medially centred concavity, similar to those of *Pe. xanthopus*, *Do. luctuosa*, *Do. muelleri* and *B. lesueur*. The trochlea is deeper than in *M. fuliginosus* and *Po. tridactylus*, shallower than in *No. eugenii* and *L. fasciatus*, less medially displaced than in *S. brachyurus*, *De. lumholtzi* and *L. fasciatus*, and slightly shallower than in *T. billardierii*.

**Table 4.** Dimensions in mm of tali and cuboids of *Dorcopsoides fossilis.*

specimen number	talus	
	length	width
NTM P5779-1	16.9	19.4
NTM P5779-2	20.3	21.2
NTM P8828	?	22.3
NTM P5778-1	18.3	20.7
NTM P5778-2	19.2	21.8
NTM P4920	18.3	20.4
NTM P5799	18.7	20.9
NTM P8718-1	19.2	21.2
NTM P5801-1	19.3	21.2
NTM P5801-2	18.1	20.6
NTM P5801-3	18	20.7
NTM P5810	20	?
NTM P5789	?	20
NTM P4458	19.4	20.8
NTM P5788	17	19.1
NTM P5793	16.7	19.5
NTM P5786-1	18.1	20.3
NTM P5786-2	17.7	21.2
NTM P4971	18	20.9

specimen number	cuboid		
	length	width	height
NTM P4590-1	12.5	15.1	14.5
NTM P4590-2	12.8	14.9	?
NTM P4590-3	12.1	13.5	13.7
NTM P4758	11.6	14.2	13.7
NTM P5399-1	12.1	14.7	14.4
NTM P5399-2	12	14.5	13.1
NTM P5399-3	11.7	14.2	13.9
NTM P5399-4	12.9	16	15.2
NTM P4757-1	14.2	16	15.6
NTM P4757-2	11.6	15.2	13.6
NTM P4804-1	11.3	13.8	13.8
NTM P4804-2	12	14.3	14.6
NTM P4504	11.2	14.8	13.4
NTM P4805	12.4	14.5	?
NTM P4591-1	13.8	15.6	15.2
NTM P4591-2	13.3	16.2	14.8
NTM P4591-3	12.3	14.4	14
NTM P4591-4	12.5	15.5	13.6
NTM P4591-5	13	14.3	14.4
NTM P4591-6	12.9	14.2	14.4
NTM P4591-7	14.3	15.7	15.1
NTM P4591-8	11.7	13.5	13.5
NTM P4591-9	12.5	14.6	13.2
NTM P4591-10	12	14.8	14.9
NTM P4503-1	11.8	14.2	14.2
NTM P4503-2	12.7	14.7	14.2
NTM P4503-3	12.2	14.9	13.9
NTM P4503-4	13.3	16.6	15.6
NTM P4503-5	12	14	?
NTM P10956	13.3	15.4	?

The navicular facet (figure 7*i*) is tall, cranioplantarly convex and oval-shaped in cranial view. It is rotated slightly laterally in cranial view, to a similar degree as in *M. fuliginosus*, *No. eugenii*, *S. brachyurus* and *Pe. xanthopus* and considerably less than in *De. lumholtzi*. The height of the talar head is comparable to that of *M. fuliginosus*, *No. eugenii*, *S. brachyurus* and *Pe. xanthopus*, and taller and narrower than those of *De. lumholtzi*, *T. billardierii*, *L. fasciatus* and the two compared potoroids. The medial malleolar fossa is deep and slightly craniocaudally elongate (figure 7*h*), similar to those of *Pe. xanthopus*, *T. billardierii, Do. luctuosa* and *Do. muelleri*. This fossa is shallower and narrower in *M. fuliginosus*, *No. eugenii* and *S. brachyurus*, and broader and deeper in *De. lumholtzi* and *Po. tridactylus*. The medial malleolus extends craniomedially at a similar angle to those of *S. brachyurus*, *Pe. xanthopus* and *T. billardierii*, but with a gentle incline from the medial trochlear crest as in *De. lumholtzi*, rather than a distinct ‘step’. Those of *M. fuliginosus* and *No. eugenii* are more cranially extensive, those of *Do. luctuosa*, *Do. muelleri* and *L. fasciatus* are more medially projected, and those of the two compared potoroids are a less distinct process. A small, indistinct, craniocaudally short facet is present on the craniolateral margin of the navicular head for articulation with the cuboid (figure 7*i*), similar to those of *S. brachyurus*, *Do. luctuosa*, *Do. muelleri* and *L. fasciatus*. This facet is craniocaudally longer and dorsoplantarly shorter in *M. fuliginosus*, *No. eugenii*, *Pe. xanthopus*, *T. billardierii* and *Du. vanheurni*. The facet is absent in *De. lumholtzi*, *B. lesueur* and *Po. tridactylus*.

#### 3.2.16. Calcaneus

The calcaneal tuber of *Dd. fossilis* is approximately 1.5 times the length of the calcaneal head, and broadens gently to the caudal epiphysis (figure 7*e* and table 5), most similar to those of *T. billardierii*, *Du. vanheurni* and *L. fasciatus*. The calcaneal tuberosities of *M. fuliginosus* and *Pe. xanthopus* are relatively elongate and broaden only slightly, immediately before the caudal end, that of *Ha. puckridgi* broadens slightly less than in *Dd. fossilis* and those of *B. lesueur* and *Po. tridactylus* broaden to a greater degree towards their caudal end. *Dendrolagus lumholtzi*, *Do. luctuosa* and *Do. muelleri* have a more robust tuber that is broader close to the calcaneal head. That of *Ng. archeri* is of similar shape but is much smaller relative to the calcaneal head. The plantar surface tapers cranially such that it is triangular in shape, differing from the sub-rectangular plantar surfaces of *M. fuliginosus* and *Pe. xanthopus*.

**Table 5.** Dimensions in mm of calcanei of *Dorcopsoides fossilis*.

specimen number	calcaneus				
	craniocaudal length	calcaneal head width (sustentaculum tali to fibular facet)	calcaneal tuber depth	**calcaneal tuber width**	width of talar facets
NTM P4904-1	42	23.5	?	?	17.7
NTM P4904-2	43	23.8	15.6	13.9	18.3
NTM P4904-3	?	20.8	?	?	16.2
NTM P4904-4	?	21.2	?	?	16.8
NTM P4904-5	?	?	?	?	19.4
NTM P4904-6	?	21.7	?	12.6	16.5
NTM P4904-7	?	20.7	?	?	16.2
NTM P4904-8	?	21.9	?	?	17.5
NTM P4904-B	?	?	13.6	13.8	?
NTM P4904-C	40.9	22.3	14.6	14.2	16.7
NTM P4905	39.5	21.7	14.1	13.2	17.6
NTM P4921	39.9	23	15.4	?	18.1
NTM P878-15	41.3	22.2	?	?	16.5
NTM P6012	44	24	15.7	14.2	18.2
NTM P4619	38.8	22.2	?	14.1	17.3
NTM P6011	39.9	20.6	13.8	13.6	17.3
NTM P878-2	42.3	22.7	?	13.8	16.7
NTM P878-3	40.2	21.2	12.5	12.8	17.1
NTM P878-12	40.6	21.9	13.9	13.3	17
NTM P4502	38.3	20.6	12.4	12.4	16.1
NTM P4617	39.4	21.8	?	13.2	17.2
NTM P878-21	39.6	?	14.3	13.4	17
NTM P4621	40.5	?	15.1	14.7	16.9
NTM P878-17	36.9	21.5	13.5	13.3	16.3
NTM P878-18	?	19.5	?	?	15.4
NTM P878-19	35.9	?	?	12.5	?
NTM P878-5	43.7	21.4	?	?	18.6
NTM P878-6	41.4	22.4	13.4	13.3	17.5
NTM P878-7	38.6	20.2	?	13	16.1
NTM P18449	38	21.3	13.1	12.8	17
NTM P18450	38.8	22.2	13.4	13.8	17.2
NTM P10955	42.8	24.4	?	?	17.4

The calcaneal head is broad, with a large lateral talar facet and large, medially projected sustentaculum tali, most similar to those of *T. billardierii* and *Du. vanheurni*. The calcaneal heads of *M. fuliginosus*, *No. eugenii* and *Ha. puckridgi* are narrower relative to the width of the tuber, and those of *Ng. archeri*, *B. lesueur* and *Po. tridactylus* are relatively much broader and are medially displaced relative to the calcaneal tuber. The sustentaculum tali (which directs the tendons of the m. flexor digitorum profundus and m. flexor digitorum superficialis) [86] is rounded in medial view, and projects medially beyond the margin of the medial talar facet (figure 7*a*). It projects to a similar degree to in *T. billardierii*, *De. lumholtzi*, *Du. vanheurni*, *Do. luctuosa*, *Do. muelleri* and *Po. tridactylus*, much more than in *M. fuliginosus* and *Ha. puckridgi*, more than in *No. eugenii*, *Pe. xanthopus* and *L. fasciatus*, and less than in *S. brachyurus*, *Ng. archeri* and *B. lesueur*. The sustentaculum tali is more pointed in medial view in *Do. luctuosa* and *Do. muelleri*. The fibular facet (figure 7*a*) is most similarly shaped to those of *S. brachyurus* and *Du. vanheurni* but differs from *S. brachyurus* in being projected further laterally with a larger, more rounded lateral notch. The fibular facets of *M. fuliginosus*, *No. eugenii*, *Do. luctuosa* and *Do. muelleri* are broader, while those of *De. lumholtzi* and *Ng. archeri* are narrower. The lateral talar facet is large, moderately convex and tapers gently in craniocaudal length medially. It is similar in shape to those of *Pe. xanthopus*, *T. billardierii*, *Du. vanheurni*, *Do. luctuosa*, *Do. muelleri* and *Ha. puckridgi* but is less bulbous dorsally than all these bar *Ha. puckridgi*. Those of *M. fuliginosus* and *No. eugenii* are not tapered medially, while those of the two potoroids are relatively smaller and more caudally situated. That of *L. fasciatus* is smaller, tapers less and is rotated craniomedially. The medial talar facet is oval, oriented obliquely from caudolateral to craniomedial, gently concave, moderately cranially tilted, and situated slightly caudally relative to the lateral talar facet. That of *Du. vanheurni* is most similarly shaped, with those of *No. eugenii* and *Pe. xanthopus* being narrower, and those of *T. billardierii*, *Do. luctuosa* and *Do. muelleri* being more rounded and larger relative to the lateral talar facet. The medial talar facet of *M. fuliginosus* and *L. fasciatus* is broader and more cranially tilted, that of *De. lumholtzi* is relatively larger, those of *De. lumholtzi* and *Do. muelleri* are more cranially situated relative to the lateral talar facet, and those of the two potoroids are relatively larger, more rounded and less cranially tilted.

The step between the dorsomedial and dorsolateral cuboid facets is distinct and slightly bevelled (has a sloping edge) (figure 7*a,f*), most similar to that of *T. billardierii*. The step is more bevelled and less pronounced than in *M. fuliginosus*, *No. eugenii*, *Pe. xanthopus* and *L. fasciatus*, slightly less bevelled than in *S. brachyurus* and *Du. vanheurni*, and less bevelled than in *De. lumholtzi*, *Do. luctuosa*, *Do. muelleri*, *Ng. archeri* and the two compared potoroids*.* The plantar margin of the plantomedial cuboid facet is at the level of the plantar surface of the calcaneal tuber, as seen in *No. eugenii*, *S. brachyurus*, *Du. vanheurni*, *Do. luctuosa*, *Do. muelleri* and both potoroids, not dorsally situated as in *M. fuliginosus*, *Pe. xanthopus* and *L. fasciatus*. The plantar cuboid facet is continuous with the cranially projected dorsomedial cuboid facet, curving plantarly then medially beneath a broad, variably deep fossa (figure 7*f*). This condition is the same in both compared potoroids and all compared macropodines except *De. lumholtzi*, in which the three cuboid facets are continuous. That of *L. fasciatus* differs in having the plantomedial facet laterally and medially semicontinuous with both dorsal facets.

#### 3.2.17. Cuboid

The cuboid of *Dd. fossilis* is roughly square in dorsal view and is slightly wider than it is long (figure 7*j–o* and table 4). All compared taxa except *Ha. puckridgi* and *Ng. archeri* differ from *Dd. fossilis* in having cuboids that are wider relative to their length. A large, moderately deeply concave surface covers most of the medial surface between the medially projecting cranial and caudal margins (figure 7*l*). The caudodorsal margin of the medial surface of the cuboid has a tall, very narrow (craniocaudally short) facet for the talar head, semicontinuous with a long, approximately oblong facet for the navicular across the centre of the dorsal half of the medial fossa. The main facet for the ectocuneiform is tall, variably oblong, and abuts the cranial margin, extending plantarly nearly to the base of the medial plantar tubercle. A small, rounded facet for articulation with the plantar tubercle of the ectocuneiform is present on the medial surface of the medial plantar tubercle. The talar facet, navicular facet and medial fossa are most similar in *S. brachyurus*, *Du. vanheurni*, *Do. luctuosa* and *Do. muelleri*. The medial fossa is shallower in *M. fuliginosus* and *Pe. xanthopus. Macropus fuliginosus* and *No. eugenii* have two small, separate navicular facets, one abutting the talar facet and one in the centre of the medial fossa, with all three of these facets smaller and less distinct in *No. eugenii*. The main ectocuneiform facet is similar to those of *S. brachyurus*, *T. billardierii*, *Du. vanheurni*, *Do. luctuosa* and *Do. muelleri*. This facet is split into two or three smaller, slightly more concave facets in *M. fuliginosus*, is smaller and less distinct in *No. eugenii* and *Pe. xanthopus*, larger and semicircular in *De. lumholtzi*, and elongate and dorsoplantarly shorter in *B. lesueur* and *Po. tridactylus. Dendrolagus lumholtzi* lacks a talar facet, has a more caudally restricted navicular facet and a larger, deeper ectocuneiform facet, and *De. lumholtzi* and *Po. tridactylus* lack a facet for the plantar tubercle of the ectocuneiform. In *L. fasciatus* and *B. lesueur* this plantar facet for the ectocuneiform is situated on the medial surface of the process for the plantar calcaneal facet.

The lateral plantar tuberosity (figure 7*k,n*) is large, fairly broad and oval in plantar view, and extends caudolaterally to beneath the plantar calcaneal facet. The shape and degree of plantar projection is variable, but is overall most similar to that seen in *Du. vanheurni* and *Do. luctuosa*. The lateral plantar tuberosity in *M. fuliginosus* and *No. eugenii* is more plantarly projected and craniocaudally elongate, and those of *S. brachyurus* and *Pe. xanthopus* are similarly projected but broader, extending craniolaterally up the side of the cuboid to the margin of the dorsolateral calcaneal facet. Those of *De. lumholtzi* and the two potoroids are less projected, with *De. lumholtzi* having a much craniocaudally shorter tuberosity. That of *Ha. puckridgi* is more rounded and larger in plantar view, though similarly plantarly projecting, and that of *L. fasciatus* is more medially situated. The medial plantar tubercle is rounded in plantar view, and is plantarly projected, generally subequal to the lateral plantar tuberosity. Those of *S. brachyurus*, *Do. luctuosa* and *Do. muelleri* are similarly shaped but less plantarly projecting; those of *M. fuliginosus*, *No. eugenii* and *Pe. xanthopus* are similarly projected but much more elongate, and those of *De. lumholtzi* and *Ha. puckridgi* are much smaller and more elongate. The medial plantar tubercle is very reduced in *Du. vanheurni* and the two potoroids and is absent in *L. fasciatus*. The flexor groove between the two plantar processes (figure 7*k*), through which passes the thick tendon of the m. flexor digitorum profundus [86,87], is narrow and moderately deep. It is proportionally similar in width to those of *M. fuliginosus*, *No. eugenii*, *S. brachyurus*, *Do. luctuosa*, *Do. muelleri* and *Ha. puckridgi*, and narrower than in *Pe. xanthopus*. In *De. lumholtzi* and the two potoroids the groove is broad and very shallow. In *L. fasciatus* this groove is formed between the lateral plantar tuberosity of the cuboid and the plantar tubercle of the ectocuneiform.

The metatarsal IV facet is wider than its height, as in all compared taxa. The metatarsal V facet is situated plantolaterally on the distal face of the cuboid and is semicontinuous with dorsal facet for metatarsal IV (figure 7*n*), though the facets are more discrete from one another than in *S. brachyurus*. In *B. lesueur* and *Po. tridactylus* the metatarsal V facet is smaller and gently convex. The plantar component of the metatarsal IV facet, which articulates with the proximoplantar process of the metatarsal IV, is variable in *Dd. fossilis*; it is relatively well developed and continuous with the dorsal facet in some specimens (e.g. NTM P4805) and very reduced (see figure 7*n*) or absent in others (e.g. NTM P4951). The absence of this plantar facet was not observed in any comparative species, though in *De. lumholtzi* the plantar facet is reduced and almost indistinguishable from the dorsal component, and in *L. fasciatus*, *B. lesueur* and *Po. tridactylus* the plantar facet is considerably broader but less plantarly extensive. The plantar facet is separate from the dorsal facet in some specimens of *M. fuliginosus*, *No. eugenii* and *Pe. xanthopus*, which was not observed in *Dd. fossilis*.

#### 3.2.18. Navicular

**Figure 8.**
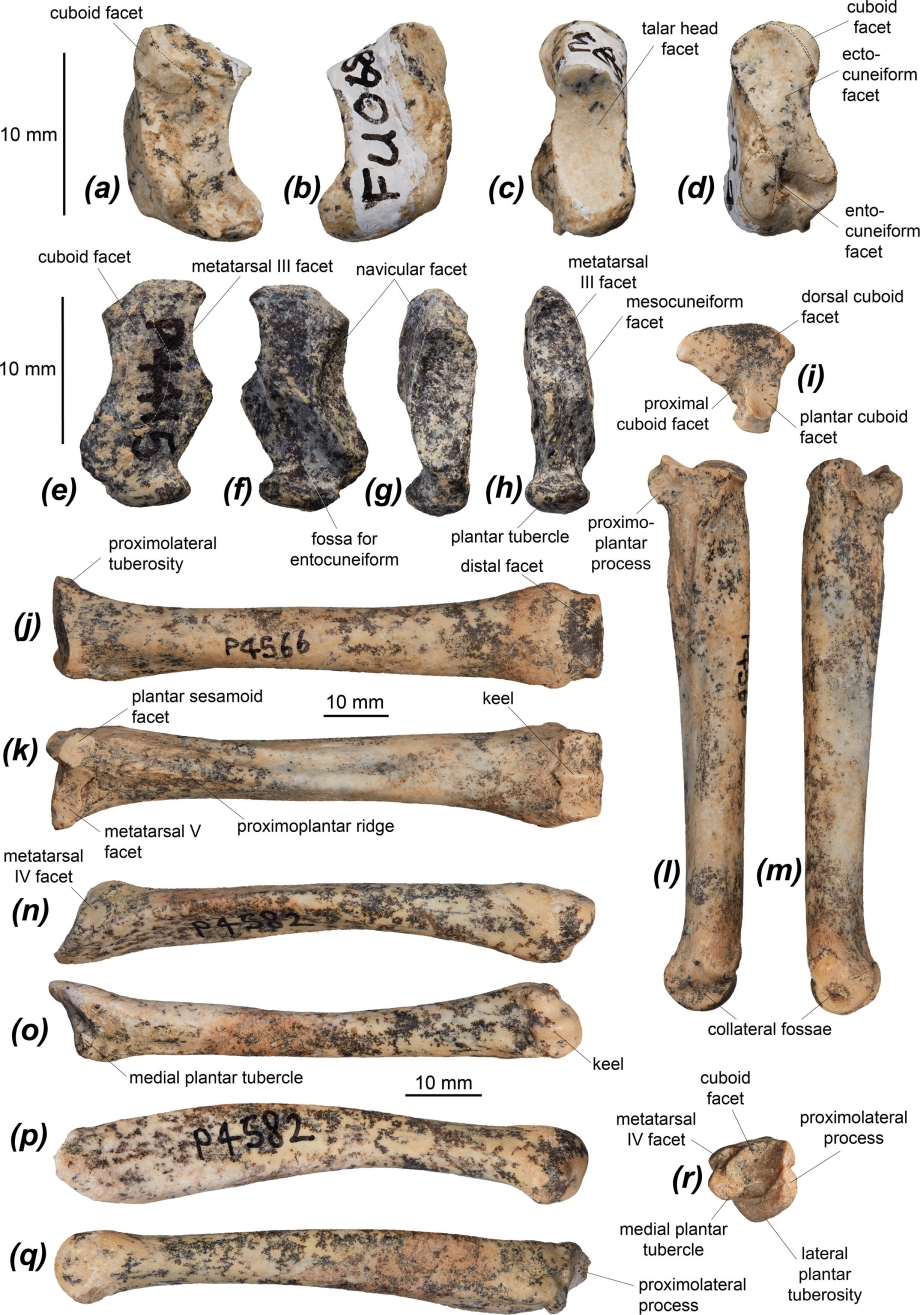
Pedal elements of *Dorcopsoides fossilis*: (*a*–*d*) left navicular NTM P18451 in (*a*) lateral, (*b*) medial, (*c*) proximal and (*d*) distal views; (*e*–*h*) right ectocuneiform NTM P4415 in (*e*) medial, (*f*) lateral, (*g*) proximal and (*h*) distal views; (*i*–*m*) left metatarsal IV NTM P4566 in (*i*) proximal, (*j*) dorsal, (*k*) plantar, (*l*) medial and (*m*) lateral views; and (*n*–*r*) right metatarsal V NTM P4582 in (*n*) dorsal, (*o*) plantar, (*p*) lateral, (*q*) medial and (*r*) proximal views.

The navicular of *Dd. fossilis* is known from six near-complete and complete specimens (figure 8*a–d*). It is short and fairly broad, most similar in overall proportions to those of *T. billardierii*, *Du. vanheurni*, *Do. luctuosa*, *Do. muelleri*, *Ha. puckridgi*, *L. fasciatus* and *G. robustiter*. Those of *M. fuliginosus*, *No. eugenii* and *Pe. xanthopus* are slightly taller and narrower, that of *S. brachyurus* is considerably taller, that of *De. lumholtzi* is shorter and much broader, and those of *B. lesueur* and *Po. tridactylus* are slightly broader. The talar facet covers the caudal surface and is smoothly, deeply concave, with similar concavity to in *T. billardierii*, *Pe. xanthopus* and *L. fasciatus*. A caudally projected plantar section makes those of *S. brachyurus*, *Ha. puckridgi*, *B. lesueur* and *Po. tridactylus* more deeply concave, while *M. fuliginosus*, *No. eugenii*, *De. lumholtzi*, *Du. vanheurni*, *Do. luctuosa*, *Do. muelleri* and *G. robustiter* have a less concave talar facet. Covering the dorsal four-fifths of the cranial surface is a tall, narrow ectocuneiform facet, with a broad, rounded, gently concave dorsal section that narrows plantarly then plantolaterally to a point, becoming slightly convex in its plantar half. Plantomedially situated on the cranial surface is a small, narrow, slightly convex facet for the entocuneiform (figure 8*d*). The entocuneiform and ectocuneiform facets are semicontinuous, delimited by a very slight furrow plantar to a gently raised seam. These two facets are most similar to those of *Du. vanheurni*, *Do. luctuosa* and *Do. muelleri*, differing from the former in having a narrower plantar section of the ectocuneiform facet, and *Ha. puckridgi*, differing in having more discrete facets. The two facets are taller and narrower and cover the whole of the cranial surface in *M. fuliginosus* and *No. eugenii*. In *S. brachyurus* and *T. billardierii* the ectocuneiform facet is broader and less extensive plantarly, with *T. billardierii* having a very small, narrow secondary ectocuneiform facet present plantolateral to the main one. In *S. brachyurus* and *Pe. xanthopus* the ectocuneiform and entocuneiform facets are more discrete, and more separate again in *L. fasciatus* and *Po. tridactylus*. In *De. lumholtzi* the ectocuneiform facet is much broader and dorsally situated and is continuous with a much taller entocuneiform facet. In *L. fasciatus*, *B. lesueur* and *Po. tridactylus* the plantar section of the ectocuneiform facet wraps around onto the lateral surface.

#### 3.2.19. Ectocuneiform

The ectocuneiform is larger relative to the size of the cuboid in *Dd. fossilis* than in the compared macropodines, similar to *Ha. puckridgi* and *L. fasciatus*, and relatively smaller than in *B. lesueur* and *Po. tridactylus* (figure 8*e–h*). The ectocuneiform is most similar in proportions and morphology to that of *Du. vanheurni*. The navicular facet is tall, fairly narrow, moderately concave, with rounded dorsal and plantar margins, and covers the dorsal three-quarters of the caudal surface (figure 8*f,g*). It is gently laterally bowed in caudal view. Those of *T. billardierii*, *Do. luctuosa* and *Do. muelleri* are most similar, though all less laterally bowed. Those of *M. fuliginosus*, *No. eugenii* and *Pe. xanthopus* are narrower and less concave, those of *S. brachyurus*, *L. fasciatus* and the two potoroids are dorsoplantarly shorter and slightly more concave, and in *De. lumholtzi* the facet is far broader and more rounded. The cuboid facet is tall, narrow and oblong (figure 8*e*), extending from near the dorsal margin to three-quarters of the distance to the plantar tubercle on the lateral surface. It is most similar to those of *Du. vanheurni*, *Do. luctuosa*, *Do. muelleri* and *Ha. puckridgi*, though in *Du. vanheurni* the facet is larger relative to the size of the ectocuneiform. In *M. fuliginosus*, *No. eugenii* and *Pe. xanthopus* the cuboid facet is separated into two smaller, more rounded facets, in *S. brachyurus* and *T. billardierii* the facet is delimited into two rounded sections by a slight seam, in *L. fasciatus* the cuboid facet covers the lateral surface of the ectocuneiform excluding the dorsalmost one-quarter and in *Po. tridactylus* it is very small and indistinct.

The plantar tubercle is rugose, well developed, elongate, slightly plantarly projected, and rounded in plantar view, with a gently to moderately convex plantar surface. It is subequal in length to the dorsal margin of the ectocuneiform, and in some specimens (e.g. NTM P6015) is slightly cranially projected. It is overall most similar to those of *S. brachyurus*, *T. billardierii*, *Du. vanheurni*, *Do. luctuosa*, *Do. muelleri* and *G. robustiter*; it differs from all these except *S. brachyurus* and *G. robustiter* in being narrower relative to length and from *T. billardierii* in having a less flattened plantar surface. *Macropus fuliginosus* and *No. eugenii* have a more plantarly projected plantar tubercle that is larger relative to the rest of the ectocuneiform, in *Pe. xanthopus* the plantar tubercle is much smaller relative to the rest of the ectocuneiform, in *De. lumholtzi* it is broader, more rounded, less rugose and less plantarly projected, in *Ha. puckridgi* the plantar tubercle is similarly sized but narrower and slightly laterally angled, in *L. fasciatus* it is narrower and has a pointed cranial eminence, and in *Po. tridactylus* the plantar tubercle is more plantocranially projected. There is a deep depression for accommodation of the entocuneiform on the medial side of the ectocuneiform (figure 8*f*), between the plantar tubercle and a thin ridge across the centre of the medial surface with a craniodorsal tilt. There is a small facet situated dorsally within the medial depression for articulation with the entocuneiform. This medial depression is similar in size and depth to those of *S. brachyurus*, *Du. vanheurni*, *L. fasciatus*, *B. lesueur* and *Po. tridactylus*, and shallower and less concave in *M. fuliginosus* and *No. eugenii*. The fossa is smaller and shallower in *De. lumholtzi*, with the facet dorsally displaced. The thin dorsal ridge is more medially prominent in *No. eugenii*, *S. brachyurus* and *Do. luctuosa*. The facet for metatarsal III is approximately D-shaped, moderately concave, and occupies the dorsal one-third to one-half of the distal surface of the ectocuneiform (figure 8*h*). The metatarsal III facet is similar in all examined species except *M. fuliginosus* and *B. lesueur*, in which it is less concave, and *Ha. puckridgi*, *G. robustiter* and *Po. tridactylus*, in which it is dorsoplantarly shorter.

#### 3.2.20. Metatarsal IV

The metatarsal IV of *Dd. fossilis* is straight and elongate (figure 8*i–m* and table 6). It is similar in gracility to those of *No. eugenii*, *Pe. xanthopus*, *Du. vanheurni*, *L. fasciatus*, *B. lesueur* and *Po. tridactylus*, more gracile than those of *S. brachyurus*, *De. lumholtzi*, *Do. luctuosa*, *Do. muelleri* and *Ng. archeri*, and more robust than those of *M. fuliginosus* and *T. billardierii*. The proximolateral tuberosity flares laterally (figure 8*j*), more so than in *M. fuliginosus* and *No. eugenii* and less so than in the two potoroids. The dorsal cuboid facet is broad, slightly medially tilted, gently convex in its lateral component and concave in its medial component, with its medial component extending plantarly to be continuous with the small, gently dorsally tilted plantar cuboid facet (figure 8*i*). It is similar in size and shape to those of all compared macropodines except *De. lumholtzi*, differing from those of *M. fuliginosus*, *No. eugenii*, *Do. luctuosa* and *Do. muelleri* in having the dorsal and plantar components continuous with each other. It differs from *De. lumholtzi* in being taller and less medially tilted, with a large concavity in the plantolateral margin and a more distinct plantar component, from *Ha. puckridgi* in having a much less convex dorsal margin, from *L. fasciatus* in being narrower and having a shorter and more plantarly situated proximal cuboid fossa, and from the potoroids in being taller and narrower. The metatarsal V facet is fairly large, slightly concave and laterally projected. It faces plantarly with a lateral tilt and a slight distal tilt (figure 8*k*), similar to that of *L. fasciatus*. The facet is oblong with a slight waist such that it is approximately figure-of-eight in shape. It does not extend plantarly onto the proximoplantar process, unlike in *M. fuliginosus*, *No. eugenii*, *Pe. xanthopus*, *Du. vanheurni*, *Do. luctuosa* and *Do. muelleri*. The metatarsal V facet is smaller in *M. fuliginosus* and *No. eugenii* with a smaller dorsal component, is much larger and more deeply concave in *De. lumholtzi*, and faces plantarly and very slightly distolaterally in *Po. tridactylus*.

**Table 6.** Dimensions in mm of fourth and fifth metatarsals of *Dorcopsoides fossilis*.

specimen number	metatarsal IV		
	length	minimum diaphyseal width	length to width index
NTM P4526	74	8.3	8.9
NTM P4298	75.7	8.7	8.7
NTM P4525	80.1	8.7	9.2
NTM P4564	78.5	8.5	9.2
NTM P876-1	76.9	9.2	8.4
NTM P4700	79.4	8.5	9.3
NTM P8775-13	78	8.2	9.5
NTM P5575-1	75.8	9	8.4
NTM P5575-2	78.5	8.6	9.1
NTM P5575-3	75.5	8.9	8.5
NTM P5575-4	77.3	8.6	9
NTM P8867	76.4	9.8	7.8
NTM P4568	74.4	8.1	9.2
NTM P4699	75.3	8.5	8.9
NTM P4566	85.6	9.3	9.2
NTM P4567	75	8.6	8.7
NTM P4565	74.4	9	8.3
NTM P4711	79.3	8.5	9.3
NTM P4692-1	78.5	8.6	9.1
NTM P4692-2	76.2	8.8	8.7
NTM P876-8	71	8.1	8.8
NTM P10961	85.7	9.4	9.2

specimen number	metatarsal V		
	length	distal epiphysis width	length to width index
NTM P4582	67.5	9.2	7.3
NTM P4581	65.3	10.1	6.5
NTM P4580	64.1	9.6	6.7
NTM P4406	73.2	11.2	6.5
NTM P6081	69.9	10	7
NTM P4406	66.2	10.1	6.6
NTM P4562	70	11	6.4
NTM P10965	?	10.8	?

The proximal dorsal surface of the metatarsal IV is gently convex, becoming flat distally, similar to all compared macropodids except *Ha. puckridgi*, in which it is more convex proximally. The diaphysis is level on its dorsal margin. *Macropus fuliginosus*, *No. eugenii*, *Pe. xanthopus* and *Ha. puckridgi* have a diaphysis that arches dorsally, a characteristic most pronounced in *Pe. xanthopus*. The proximoplantar ridge is prominent (figure 8*k*), extending one-third to two-thirds of the length of the diaphysis, and is relatively longer in larger individuals. The proximoplantar ridge is more raised in *M. fuliginosus* and *Ha. puckridgi*, similarly raised but more elongate in *No. eugenii*, less raised in *S. brachyurus*, *De. lumholtzi* and *L. fasciatus* and only extends one-third of the diaphysis, and much less raised in *B. lesueur* and *Po. tridactylus.* In dorsal view the diaphysis tapers rapidly from the proximal end to a minimum width around one-quarter of its length, then gently broadens to a maximum immediately proximal to the collateral ligament fossae on the distal epiphysis. The distal end is relatively narrower in *M. fuliginosus*, in which little waisting of the diaphysis is present, and is relatively broader in *B. lesueur*, in which the distal end appears swollen. The collateral ligament fossae on the medial and lateral surfaces of the distal epiphysis are rounded and fairly shallow, similar to those of *S. brachyurus*, *Du. vanheurni* and *Ha. puckridgi*, and shallower than in *M. fuliginosus*, *No. eugenii*, *Pe. xanthopus*, *Do. luctuosa*, *Do. muelleri*, *L. fasciatus* and the two potoroids. The distal epiphysis has a narrow, plantarly prominent plantar keel (figure 8*k*), which is subequal to more plantarly projected than the medial and lateral processes. It is very similar to those of *M. fuliginosus*, *T. billardierii*, *Pe. xanthopus*, *B. lesueur* and *Po. tridactylus*, narrower and less plantarly prominent than in *S. brachyurus* and *De. lumholtzi*, less plantarly prominent than in *No. eugenii*, *Du. vanheurni*, *Do. luctuosa*, *Do. muelleri* and *L. fasciatus*, and narrower and more plantarly prominent than in *Ha. puckridgi*.

#### 3.2.21. Metatarsal V

The metatarsal V of *Dd. fossilis* is fairly elongate and moderately mediolaterally compressed (figure 8*n–r* and table 6). It is straight to slightly distolaterally curved in dorsal view. In gracility it is similar to those of *No. eugenii*, *T. billardierii*, *Do. luctuosa*, *Do. muelleri* and *Ha. puckridgi*, more robust than in *M. fuliginosus*, *Pe. xanthopus*, *L. fasciatus* and *B. lesueur*, and more gracile than in *S. brachyurus*, *De. lumholtzi*, *Du. vanheurni* and *Po. tridactylus*. The proximolateral process is dorsoplantarly short, pointed at its tip, and projected proximally well beyond the margin of the cuboid facet (figure 8*q*,*r*). The process is similarly developed in *S. brachyurus*, *T. billardierii*, *Du. vanheurni*, *Do. luctuosa*, *Do. muelleri*, *Ha. puckridgi*, *L. fasciatus* and *Po. tridactylus*, but is more rounded in all except *T. billardierii*, *Do. luctuosa* and *Ha. puckridgi*. The cuboid facet varies from rounded to oblong (figure 8*r*) and extends medioplantarly from a small, dorsolaterally flared eminence dorsal to the proximolateral process onto the proximal base of the medial plantar tubercle. It is similar in shape and size to that of *T. billardierii*, being broader and more obliquely orientated than in all compared species except in *M. fuliginosus*, which has a similarly oblique orientation, in *De. lumholtzi*, which has a broader cuboid facet, and in *Du. vanheurni*, *Do. luctuosa* and *Do. muelleri*, in which the facet is similarly broad. The metatarsal IV facet (figure 8*o*,*r*) is broad, transversely orientated with a slight proximolateral rotation, and mirrors the cuboid facet on the metatarsal IV in being figure-of-eight shaped. The metatarsal IV facet is separated from the cuboid facet, not semicontinuous as in *De. lumholtzi*, *Do. luctuosa*, *Do. muelleri* and the two potoroids, nor abutting as in *S. brachyurus*, *T. billardierii*, *Du. vanheurni* and *Ha. puckridgi*. The lateral plantar tuberosity is rugose, raised, fairly narrow and elongate, extending one-third to half of the length of diaphysis, and is bounded by a deep, narrower sulcus running posteromedially. This tuberosity is similarly raised in *T. billardierii*, *Do. luctuosa* and *Do. muelleri*, but is less elongate in *T. billardierii*. That of *Ha. puckridgi* is similarly elongate but more raised, and that of *L. fasciatus* is more raised but less elongate. The medial plantar tubercle is small but distinct, rounded to oblong, and medioplantarly projected. This tubercle is similarly developed in *S. brachyurus*, smaller in *T. billardierii*, *Du. vanheurni*, *Do. luctuosa* and *Do. muelleri*, and is very small to absent in *M. fuliginosus*, *No. eugenii*, *Pe. xanthopus*, *De. lumholtzi*, *Ha. puckridgi* and *L. fasciatus*. In *B. lesueur* and *Po. tridactylus* the medial plantar tubercle is very large, with *Po. tridactylus* possessing a rounded facet for articulation with the proximal plantar sesamoid and to a smaller extent with the proximoplantar process of metatarsal IV (figure 8*l*).

The metatarsal V is very slightly arched dorsally, less so than in *M. fuliginosus*, *No. eugenii*, *S. brachyurus*, *T. billardierii*, *De. lumholtzi* and *Po. tridactylus*, most similar to *Pe. xanthopus*, *Du. vanheurni*, *Do. luctuosa* and *Do. muelleri*, and more so than in *L. fasciatus* and *B. lesueur*, which lack any diaphyseal arch. The diaphysis is similarly transversely compressed to that of *Pe. xanthopus*, and less so than in *M. fuliginosus*, *No. eugenii*, *Pe. xanthopus* and *Ha. puckridgi*. In cross-section the diaphysis approaches triangular, particularly in its proximal half, similar to the compared dorcopsins. *Setonix brachyurus*, *De. lumholtzi* and *Po. tridactylus* have only slight medial flattening of the diaphysis. The distal epiphysis is fairly small relative to the dimensions of the diaphysis, with a larger medial component and smaller, slightly laterally projected lateral component. The proportions of the epiphysis are similar to those of *No. eugenii* and *Pe. xanthopus*, being broader relative to height than in *M. fuliginosus* and relatively narrower than in the other compared species. The narrow keel (figure 8*o*) is slightly more plantarly prominent than the medial and lateral crests, similar to *M. fuliginosus* and *Ha. puckridgi*, and less prominent than in the remaining compared species. The collateral fossae either side of the distal epiphysis are subequally shallow and rounded, similar to those of *Ha. puckridgi*. In *M. fuliginosus* these fossae are larger and deeper, in *No. eugenii*, *S. brachyurus*, *Pe. xanthopus*, *L. fasciatus*, *B. lesueur* and *Po. tridactylus* just the medial collateral fossa is larger and deeper, in *De. lumholtzi* they are both larger, and in *Du. vanheurni*, *Do. luctuosa* and *Do. muelleri* they are deeper.

#### 3.2.21.1. Proximal pedal phalanx IV

**Figure 9.**
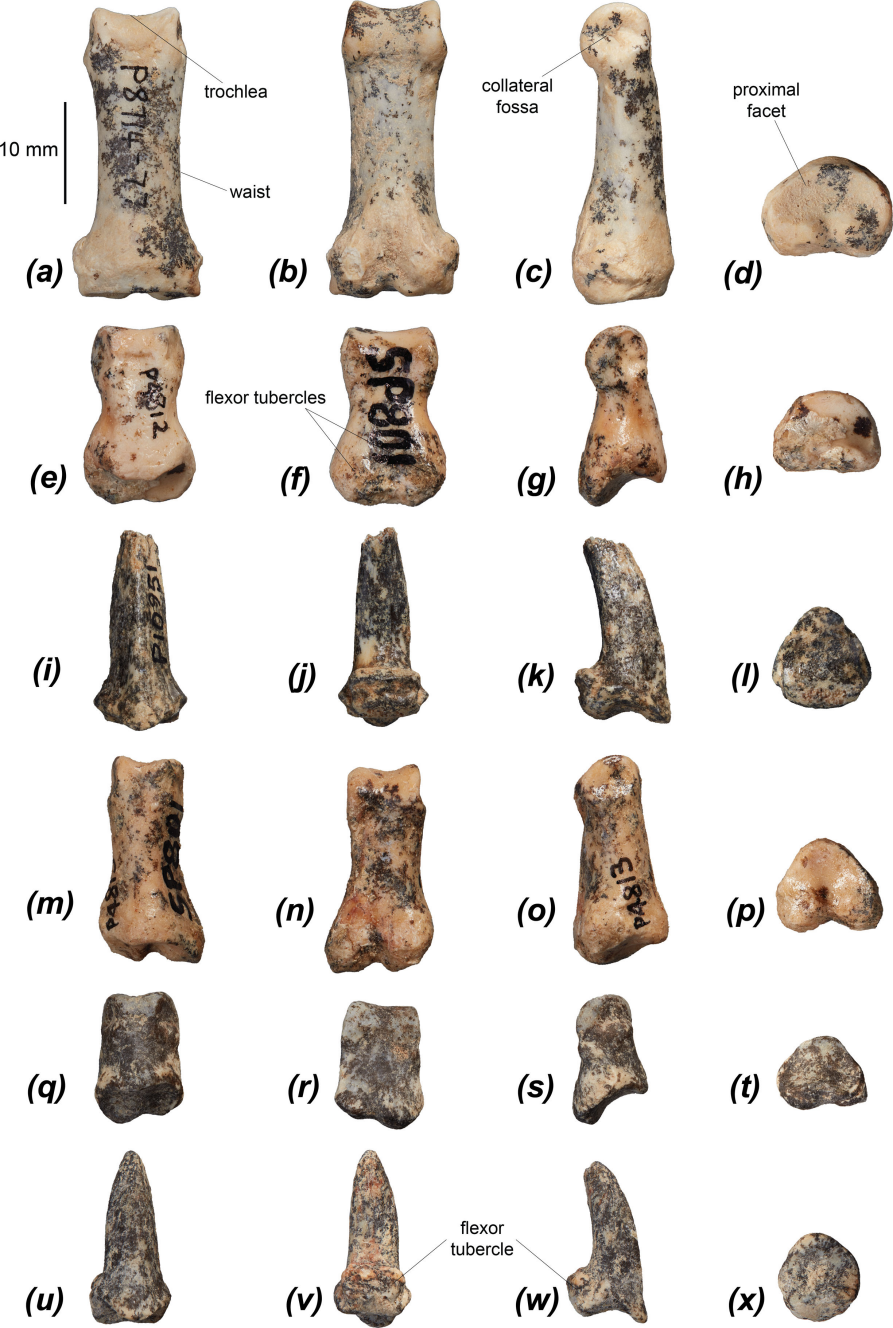
Pedal phalanges of *Dorcopsoides fossilis* in (left to right) dorsal, plantar, left transverse and proximal views: (*a*–*d*) left proximal phalanx IV NTM P8714-77; (*e*–*h*) left middle phalanx IV NTM P4812; (*i*–*l*) right distal phalanx IV NTM P10951; (*m*–*p*) left proximal phalanx V NTM P4813; (*q*–*t*) right middle phalanx V NTM P18452; and (*u*–*x*) right distal phalanx V NTM P18454.

The proximal pedal phalanx IV of *Dd. fossilis* is very well represented, known from 27 complete and near-complete specimens (figure 9*a–d* and table 7). It is elongate, with the proximal end considerably broader and the distal end slightly broader than the diaphysis, most similar in its proportions to those of *No. eugenii* and *Du. vanheurni*. It has a more distinct waist than in *M. fuliginosus*, is slightly more gracile than in *S. brachyurus*, *T. billardierii*, *Do. luctuosa* and *Do. muelleri*, is more robust with a relatively narrower distal end than in *Pe. xanthopus* and *Ha. puckridgi*, more robust than in *L. fasciatus*, slightly more robust than in *B. lesueur*, and much more robust than that of *Po. tridactylus*. The proximal (metatarsal IV) facet is reniform, gently concave and dorsoplantarly compressed, most like those of *S. brachyurus*, *Du. vanheurni*, *Do. luctuosa* and *Do. muelleri*. Those of *Pe. xanthopus*, *De. lumholtzi* and *Ha. puckridgi* are more dorsoplantarly compressed, those of *M. fuliginosus*, *No. eugenii* and *T. billardierii* are less dorsoplantarly compressed, and that of *Po. tridactylus* is slightly more concave. The trochlea is smoothly, moderately concave, most similar to those of *M. fuliginosus*, *T. billardierii*, *Do. luctuosa*, *Do. muelleri* and *Po. tridactylus*. The trochlea is slightly more concave in *No. eugenii* and *L. fasciatus*, slightly less concave in *S. brachyurus* and *Du. vanheurni*, and more concave and slightly triangular in *De. lumholtzi* and *B. lesueur*. That of *Pe. xanthopus* is flat.

**Table 7.** Dimensions in mm of fourth and fifth pedal phalanges of *Dorcopsoides fossilis*.

specimen number	element	length	proximal width
NTM P8714-93	proximal phalanx IV	29.9	14.6
NTM P8714-78	proximal phalanx IV	29	12.8
NTM P8714-77	proximal phalanx IV	30.8	13.5
NTM P8714-84	proximal phalanx IV	28.1	13
NTM P8714-49/58	proximal phalanx IV	31.8	14.1
NTM P8855	proximal phalanx IV	32.4	13.8
NTM P5564	proximal phalanx IV	28.9	13.4
NTM P5527	proximal phalanx IV	30.1	13.8
NTM P5529	proximal phalanx IV	29.5	13.3
NTM P5468	proximal phalanx IV	33.1	14.9
NTM P5467	proximal phalanx IV	30.1	13.6
NTM P5494-1	proximal phalanx IV	30.2	14.1
NTM P5494-2	proximal phalanx IV	29.7	14.2
NTM P5494-3	proximal phalanx IV	29.8	13.5
NTM P5466	proximal phalanx IV	33.5	15.3
NTM P5491-1	proximal phalanx IV	31.3	13.6
NTM P5491-2	proximal phalanx IV	32.2	13.3
NTM P5513	proximal phalanx IV	29.3	13.3
NTM P5590	proximal phalanx IV	30.9	13.7
NTM P5596	proximal phalanx IV	30.6	14.3
NTM P4996	proximal phalanx IV	29	13.2
NTM P4806-1	proximal phalanx IV	?	12.8
NTM P4806-2	proximal phalanx IV	31.2	13.2
NTM P10962	proximal phalanx IV	32.5	14.1
NTM P10963	middle phalanx IV	20.2	12.2
NTM P4812	middle phalanx IV	18	11.1
NTM P10968	distal phalanx IV	16.5	7.5
NTM P10879	distal phalanx IV	20.4	8.6
NTM P10951-1	distal phalanx IV	?	9.7
NTM P10951-2	distal phalanx IV	22	8.1
NTM P8762-1	distal phalanx IV	17.2	9.2
NTM P8762-2	distal phalanx IV	?	8.4
NTM P8762-3	distal phalanx IV	?	8.2
NTM P4813	proximal phalanx V	20.7	10.9
NTM P4814	proximal phalanx V	19.8	9.8
NTM P10967	middle phalanx V	12.8	9.9
NTM P18452	middle phalanx V	12.3	8.4
NTM P18453	middle phalanx V	10	7.6
NTM P18454	distal phalanx V	16.8	7.8
NTM P8762-4	distal phalanx V	17.8	8.3
NTM P8762-5	distal phalanx V	16.6	8.1
NTM P8762-6	distal phalanx V	17.6	7.7
NTM P10964	distal phalanx V	18.2	8.8

#### 3.2.22. Middle pedal phalanx IV

The middle fourth pedal phalanx of *Dd. fossilis* is known from two specimens (figure 9*e–h* and table 7). It is fairly robust and dorsoplantarly compressed, with a smooth, moderate waist at the midpoint of its diaphysis. It is most similar to those of *S. brachyurus*, *Du. vanheurni* and *Do. luctuosa*, differing from *S. brachyurus* in being more dorsoplantarly compressed and having a more distinct waist, and from *Du. vanheurni* in having the distal end subequal in width to the proximal end (rather than relatively narrower). The middle phalanx IV is more robust than those of *M. fuliginosus*, *No. eugenii*, *L. fasciatus*, *B. lesueur* and *Po. tridactylus*, much more robust than in *Pe. xanthopus*, and less robust than those of *Do. muelleri* and *Ha. puckridgi*. The proximal facet is reniform, smoothly, moderately concave and gently dorsally tilted. The dorsal margin of the proximal end is broad, smoothly rounded and gently dorsoproximally projected, unlike the narrower, more pointed and more projected dorsal margin in *M. fuliginosus*, *No. eugenii*, *Pe. xanthopus*, *De. lumholtzi* and *L. fasciatus*, which forms a more concave proximal facet in those species. The flexor (plantar) tubercles, for the insertion of the tendons of the m. flexor digitorum superficialis [86,87], are low, broad and rounded with a very shallow mesial groove; very similar in *S. brachyurus* and *Du. vanheurni*. These tubercles are slightly narrower in *M. fuliginosus* and *No. eugenii*, narrower and more raised in *T. billardierii* and *L. fasciatus*, lower and narrower in *Pe. xanthopus* and *De. lumholtzi*, broader in *Do. luctuosa* and *Ha. puckridgi*, and broader and more raised in *B. lesueur* and *Po. tridactylus*. In dorsal view, the proximal end of the middle phalanx has smoothly convex transverse margins giving it a rounded appearance, dissimilar to *Pe. xanthopus*, which has linear transverse margins narrowing distally, and to *De. lumholtzi*, which has only very slight transverse convexity at its proximal end.

Immediately proximodorsal to the trochlea is a shallow, approximately rectangular fossa (figure 9*e*), similar in size and depth to those of *Do. luctuosa* and *Do. muelleri*. This fossa is present in all compared species, but is much smaller and shallower in *M. fuliginosus*, *No. eugenii*, *S. brachyurus* and *T. billardierii*, shallower in *Pe. xanthopus* and *Du. vanheurni*, and larger and deeper in *De. lumholtzi* and *L. fasciatus*. The collateral fossae are large and very shallow, most similar to those of *S. brachyurus*, *T. billardierii*, *Du. vanheurni* and *Ha. puckridgi*, though slightly shallower. The collateral fossae are deeper in *M. fuliginosus*, *No. eugenii*, *De. lumholtzi*, *Do. luctuosa*, *Do. muelleri* and the two potoroids, and smaller in *Pe. xanthopus*. The trochlea is broad and gently concave, most similar to those of *S. brachyurus*, *T. billardierii*, *Do. luctuosa*, *Do. muelleri*, *Ha. puckridgi* and *L. fasciatus*. It is narrower in *M. fuliginosus*, *No. eugenii* and *Du. vanheurni*, and deeper and more triangular in *Pe. xanthopus*, *De. lumholtzi*, *B. lesueur* and *Po. tridactylus*.

#### 3.2.23. Distal pedal phalanx IV

The distal fourth phalanx of *Dd. fossilis* is known from seven specimens (figure 9*i–l* and table 7). It is fairly elongate and slightly taller than it is wide. It is most similar in proportions to those of *M. fuliginosus*, *S. brachyurus* and *Du. vanheurni*. This phalanx is slightly more gracile than those of *No. eugenii*, *Pe. xanthopus*, *Do. luctuosa* and *Do. muelleri*, much longer and more gracile than those of *T. billardierii* and *Ha. puckridgi*, more robust than that of *L. fasciatus*, and shorter and more robust than those of *De. lumholtzi*, *B. lesueur* and *Po. tridactylus*. The proximal facet is domed in proximal view and moderately concave, most similar to those of *Du. vanheurni*, *Do. luctuosa* and *Do. muelleri*. The proximal facet has a dorsal point in *M. fuliginosus*, *No. eugenii*, *S. brachyurus*, *L. fasciatus* and *B. lesueur*, is less concave in *Pe. xanthopus*, and is broader and more rounded dorsally in *T. billardierii*, *Ha. puckridgi* and *Po. tridactylus*. That of *De. lumholtzi* is more concave and has a slight mesial ridge running dorsoplantarly. The flexor tubercle is broad, proximodistally short, approximately rectangular in plantar view, and plantarly projected, similar to those of *M. fuliginosus*, *Du. vanheurni*, *Do. luctuosa* and *Do. muelleri*. The flexor tubercle is more rounded in *S. brachyurus*, less plantarly projected in *No. eugenii*, *T. billardierii* and *Ha. puckridgi*, and narrower, more elongate and less plantarly projected in *De. lumholtzi*, *Pe. xanthopus*, *L. fasciatus*, *B. lesueur* and *Po. tridactylus*.

The diaphysis is rounded-triangular in cross-section, most similar to those of *Du. vanheurni* and *Po. tridactylus*. The diaphysis in cross-section is more triangular in *M. fuliginosus*, *No. eugenii*, *S. brachyurus*, *Pe. xanthopus*, *L. fasciatus* and *B. lesueur*, more rounded in *De. lumholtzi*, *T. billardierii*, *Do. luctuosa* and *Do. muelleri*, and much more rounded in *Ha. puckridgi*. The diaphysis curves gently but distinctly plantarly towards the tip, to a similar degree as in *De. lumholtzi* and *T. billardierii*. The diaphysis is slightly less plantarly curved in *Du. vanheurni*, *Do. luctuosa*, *Do. muelleri* and *Po. tridactylus*, more plantarly curved in *Ha. puckridgi*, while those of *M. fuliginosus*, *No. eugenii*, *S. brachyurus*, *L. fasciatus* and *B. lesueur* have very slight or no plantar curvature. That of *Pe. xanthopus* arches gently dorsally then curves plantarly. The distal tip comes to a gently rounded point, similar to those of *S. brachyurus*, *Pe. xanthopus*, *Du. vanheurni*, *Do. luctuosa* and *Do. muelleri*. The tip is more strongly pointed in *M. fuliginosus*, *No. eugenii*, *De. lumholtzi*, *T. billardierii*, *L. fasciatus*, *B. lesueur* and *Po. tridactylus*, and more rounded in *Ha. puckridgi*.

#### 3.2.24. Proximal pedal phalanx V

The proximal fifth pedal phalanx of *Dd. fossilis* is known from three specimens (figure 9*m–p* and table 7). It is fairly elongate, and subequal in width and height, with a gently laterally skewed proximal end and a slight waist on the diaphysis. It is very similar to those of *Du. vanheurni* and *Po. tridactylus*, differing from the former only in having a slightly more concave trochlea and from the latter in being more robust with smaller flexor tubercles. The length of the proximal fifth phalanx is approximately two-thirds the length of the proximal fourth phalanx. The length relative to the proximal fourth phalanx is similar in *S. brachyurus*, *T. billardierii*, *Du. vanheurni*, *Do. luctuosa*, *Do. muelleri*, *L. fasciatus*, *B. lesueur* and *Po. tridactylus*. In *M. fuliginosus*, *No. eugenii* and *Pe. xanthopus* the proximal fifth phalanx is relatively smaller, in *Ha. puckridgi* it is relatively slightly larger, and in *De. lumholtzi* it is relatively much larger. The proximal facet is domed, moderately concave, and subequal in height to its width, similar to most of the compared species. Those of *M. fuliginosus* and *Pe. xanthopus* are less concave and taller relative to their width, that of *De. lumholtzi* is more deeply concave and relatively broader, and that of *L. fasciatus* is more deeply concave and narrower. The degree of proximolateral skewing is similar to those of most compared species, but is greater than in *T. billardierii*, *De. lumholtzi* and *B. lesueur*.

The diaphysis is rounded-triangular in cross-section with a slightly more convex medial side than lateral side and is most distinctly triangular at its midpoint. The diaphysis is more rounded in all compared species except *M. fuliginosus*, *No. eugenii* and *L. fasciatus*, in which it is slightly taller and more triangular. The collateral fossae do not differ from those of the proximal phalanx IV. The trochlea is gently concave, differing from those of *M. fuliginosus*, *Du. vanheurni*, *Do. luctuosa* and *Do. muelleri* in being more concave and from *De. lumholtzi* and *B. lesueur* in being less concave with a less triangular concavity.

#### 3.2.25. Middle pedal phalanx V

Three middle fifth pedal phalanges of *Dd. fossilis* are known (figure 9*q–t* and table 7). The phalanx is robust and dorsoplantarly compressed, with a short diaphysis approximately triangular in cross-section that differentiates it from all compared taxa. It is otherwise very similar to those of *S. brachyurus*, *T. billardierii*, *Du. vanheurni*, *Do. luctuosa* and *Do. muelleri*. The middle fifth pedal phalanges of *M. fuliginosus*, *No. eugenii*, *De. lumholtzi*, *Ha. puckridgi*, *L. fasciatus*, *B. lesueur* and *Po. tridactylus* are more elongate, while that of *Pe. xanthopus* is more robust and dorsoplantarly compressed. The proximal facet is strongly dorsally tilted with its medial and lateral sides gently, roundly concave. It differs from those of *M. fuliginosus*, *No. eugenii*, *Pe. xanthopus*, *De. lumholtzi*, *B. lesueur* and *Po. tridactylus* in being more dorsally tilted, and from *De. lumholtzi* in having its medial and lateral sections less medially and laterally rotated, respectively. The dorsal margin of the proximal facet is rounded and gently proximodorsally projected, similar to all compared species except *De. lumholtzi*, which has a more pointed, proximally projected dorsal margin. The plantar tubercles are low, rounded and elongated proximally beneath the proximal facet. These tubercles are smaller in *M. fuliginosus* and *De. lumholtzi*, smaller and more rounded in *Pe. xanthopus* and *Ha. puckridgi*, and slightly larger in *Po. tridactylus*.

The diaphysis narrows slightly to the distal end, with no waist. A slight waist is present on the diaphysis in *Pe. xanthopus*, *De. lumholtzi*, *Ha. puckridgi*, *L. fasciatus*, *B. lesueur* and *Po. tridactylus*. The collateral fossae are large, rounded and gently concave, larger than in *De. lumholtzi* and larger and deeper than in *Pe. xanthopus*. The trochlea is very slightly concave, less so than in *Pe. xanthopus* and *De. lumholtzi*, and is smoothly rounded rather than gently V-shaped as in *De. lumholtzi* and *L. fasciatus*. A small, variably shallow fossa abuts the centre of the dorsal margin of the distal articular surface, present and similarly variable in all compared species.

#### 3.2.26. Distal pedal phalanx V

The distal fifth pedal phalanx of *Dd. fossilis* is known from 14 specimens (figure 9*u–x* and table 7). It is elongate and slightly taller than its width at the proximal end and along the diaphysis, most similar to the middle fifth pedal phalanges of *S. brachyurus*, *T. billardierii* and *Du. vanheurni*. Those of *M. fuliginosus*, *No. eugenii* and *Pe. xanthopus* are taller, more robust and smaller relative to the distal fourth phalanx, those of *De. lumholtzi* and *Po. tridactylus* are taller, narrower and more elongate, those of *Do. luctuosa*, *Do. muelleri* and *Ha. puckridgi* are broader and more robust, and that of *L. fasciatus* is narrower. The proximal facet is rounded and moderately concave, with a rounded, dorsoproximally projected dorsal lip, similar to those of most compared species. Those of *M. fuliginosus* and *No. eugenii* have a less concave proximal facet, and that of *De. lumholtzi* has a more concave facet with a slight dorsoplantar ridge mesially and a narrower dorsal lip. *Thylogale billardierii* has a less concave facet with a similar slight mesial ridge. The flexor tubercle is well developed, broad, plantarly projected and rounded-square in plantar view, marginally narrower than the width of the proximal facet. It is most similar in shape and relative size to that of *Ha. puckridgi*. Those of *M. fuliginosus*, *No. eugenii* and *Pe. xanthopus* are larger relative to the size of the phalanx and are rounded in plantar view, that of *Pe. xanthopus* is much larger relative to the size of the diaphysis and is rounded in plantar view, that of *De. lumholtzi* is more elongate proximodistally, those of *S. brachyurus*, *T. billardierii*, *Du. vanheurni*, *Do. luctuosa* and *Do. muelleri* are slightly smaller relative to the size of the phalanx, and those of *L. fasciatus* and *Po. tridactylus* are rounded in plantar view, less plantarly projected and more elongate.

The diaphysis has a rounded-triangular dorsal peak situated just medial to the centreline of the phalanx, similar to most compared species but less angular and distinct than in *De. lumholtzi*. The diaphysis is moderately plantarly curved, with a tip that is a rounded point in dorsal view, similar to those of *Do. luctuosa* and *Do. muelleri*. Those of *M. fuliginosus*, *No. eugenii* and *Pe. xanthopus* are less plantarly curved and have a more pointed tip, those of *De. lumholtzi*, *B. lesueur* and *Po. tridactylus* are narrower and more plantarly curved with a more pointed tip, those of *T. billardierii*, *Du. vanheurni* and *L. fasciatus* are less plantarly curved, and that of *Ha. puckridgi* has a more rounded tip.

## 4. Results

### 4.1. Body-mass estimates

Following the method and using the dataset of Prideaux & Warburton [58], measurements from the calcaneus and femur of *Dorcopsoides fossilis* were used to generate mass estimates using predictive equations. Measurements were collected from 4 partial femora, 12 complete calcanei and 13 partial calcanei, producing a total of 112 mass estimates from four skeletal dimensions. The estimated range of body masses was 8.3–14.8 kg, with a mean of 10.6 kg. These were averaged across each specimen to produce 29 mass estimates with a mean of 10.9 kg and a standard deviation of 1.7. The body mass estimates are spread out with multiple weak peaks (electronic supplementary material, figure S1). Calcaneal tuber width (CTW) gives consistently lower mass estimates than other measurements, and has the lowest *r*^2^ value, 0.70. As was the case in the analysis by Prideaux & Warburton [58], femoral circumference (FC) generated slightly higher mass estimates than the calcaneal measurements. With the femoral estimates removed, the data have a loosely bimodal distribution (electronic supplementary material, figure S2).

### 4.2. Principal components analysis

**Figure 10.**
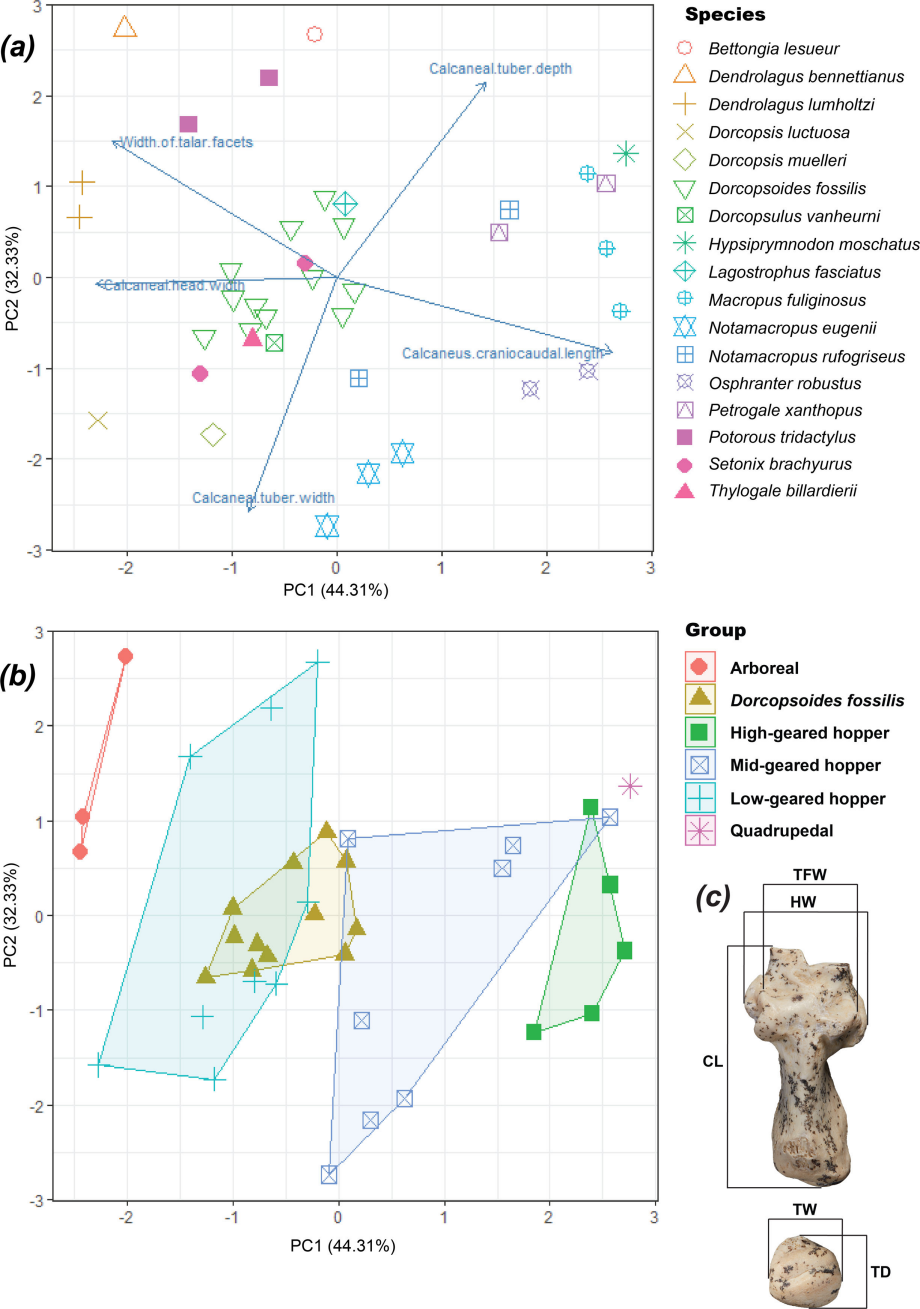
Scatterplots of PC1 versus PC2 of calcaneal measurements from *Dorcopsoides fossilis* and 16 comparative species: (*a*) scatterplot with variable loading and direction indicated by arrows and with specimen colour and shape designating species; (*b*) scatterplot with specimen shape and frame colour showing generalized ecomorphological/locomotory groups, with the fossil *Dd. fossilis* categorized as its own group; and (*c*) diagram of calcaneal measurements taken in dorsal and caudal views. Abbreviations in (*c*): TFW, talar facet width; HW, head width; CL, calcaneal length; TW, tuber width; TD, tuber depth.

A PCA was run on the five transformed calcaneal measurements from *Dorcopsoides fossilis* and comparative species. Principal component 1 (PC1) accounted for 44.31% of variation, PC2 for 32.33% and PC3 for 12.8%. A scatterplot of PC1 and PC2 (figure 10*a*) shows *Dorcopsoides fossilis* clustered centrally, occupying similar space to *Setonix brachyurus*, *Thylogale billardierii*, *Dorcopsulus vanheurni* and *Lagostrophus fasciatus. Dorcopsis luctuosa* and *Do. muelleri* are placed close to this group, below and to the left, i.e. more negative in both PC1 and PC2. *Dendrolagus lumholtzi* and *De. bennettianus* place separately from the other taxa, negative in PC1 and positive in PC2. *Bettongia lesueur* and *Potorous tridactylus* group together, approximately between the *Dd. fossilis* group and the *Dendrolagus* group. *Notamacropus eugenii* and one specimen of *No. rufogriseus* group more negative in PC2 and more positive in PC1 than the *Dd. fossilis* group. One specimen of *No. rufogriseus* places moderately positive in PC1 along with *Petrogale xanthopus*, which loosely groups with *Macropus fuliginosus* and *Osphranter robustus*, with *Hypsiprymnodon moschatus* being slightly more positive in PC1 and slightly more negative in PC2.

The loading of each variable was calculated and ordered (table 8) then figured (figure 10*a*). The three highest-loaded variables were calcaneus craniocaudal length, calcaneal head width and width of talar facets. Variables separating the arboreal group to strongly negative in PC1 and positive in PC2 and placing *Do. luctuosa* strongly negative in PC1 are the width of the talar facets and calcaneal head width. These two variables also place *S. brachyurus*, *T. billardierii*, *Do. muelleri*, *Du. vanheurni*, *Po. tridactylus* and all but three specimens of *Dd. fossilis* negatively in PC1. Calcaneal tuber width places *No. eugenii* and *Os. robustus*, one specimen each of *M. fuliginosus*, *No. rufogriseus* and *S. brachyurus*, the sole specimens of *T. billardierii*, *Do. muelleri*, *Do. luctuosa*, and roughly half the specimens of *Dd. fossilis* negatively in PC2. Calcaneal craniocaudal length places *M. fuliginosus*, a specimen of *No. rufogriseus*, *Pe. xanthopus* and *Hy. moschatus* positively in PC1, and *Os. rufus*, *No. eugenii* and the other of *No. rufogriseus* negatively in PC2 and positively in PC1. Calcaneal tuber depth acts to place *L. fasciatus*, and to a greater extent *M. fuliginosus*, *No. rufogriseus*, *Pe. xanthopus* and *Hy. moschatus* positively in both PC1 and PC2. It also acts to place *De. lumholtzi*, and to a greater extent *De. bennettianus*, *Po. tridactylus* and *B. lesueur* positively in PC2.

**Table 8.** Variable loadings from PCA of calcaneal measurements from *Dorcopsoides fossilis* and 16 comparative species. Ordered from greatest to least effect.

variable	loading
calcaneus craniocaudal length	0.595435
calcaneal head width	0.5200947
width of talar facets	0.4847177
calcaneal tuber depth	0.3229432
calcaneal tuber width	0.1889844

Figure 10*b* shows the separation of the comparative specimens into ecomorphological/locomotory groups. The arboreal group is distinct from all others, situated in the top left corner. The low-geared group covers the central-left portion of the plot, extending most of PC2. The grouped specimens of *Dd. fossilis* are just negative in PC1, overlapping chiefly with the low-geared group, covering the middle ground and overlapping to a lesser extent with the mid-geared group. The mid-geared group covers the central-right of the plot, extending to the most negative of PC2 and overlapping slightly with the high-geared group in the most positive of PC1. The high-geared group is strongly positive in PC1. The quadrupedal taxon, *Hy. moschatus*, is slightly more positive in both PC1 and PC2 than the mid-geared and high-geared groups.

## 5. Discussion

### 5.1. Body mass

The range of the body-mass estimates for *Dorcopsoides fossilis* suggests the species was heavier than living dorcopsins. The three largest extant species, *Dorcopsis hageni*, *Do. atrata* and *Do. luctuosa*, have been recorded weighing approximately 4–7 kg as adults [44,51,52,107], with the smallest species, *Dorcopsulus vanheurni*, weighing approximately 1.5–3.5 kg [51,107]. It is worth noting that there is little published body mass data for even the more common species of living dorcopsin, so the range of their body masses is likely underestimated. Anecdotal data report large male specimens of *Dorcopsis* weighing around 10 kg. If the few published data are taken as representative, *Dd. fossilis* was 1–8 kg heavier than the largest species of *Dorcopsis*.

Although speculative, this larger mass may reflect the Alcoota palaeoenvironment, which is inferred to have been more open [33,108]. Body size is linked to environmental openness in extant macropodines, with smaller species often associated with more densely vegetated habitats, and larger species with more open habitats, such as woodlands, sparse shrublands and grasslands [72,109]. This relationship is also seen in placental herbivores [110,111], such as bovids [112].

Generally small before the Late Miocene aridification of Australia, macropodids trended towards greater size over the Late Cenozoic in response to increased open habitats, lower food quality and water scarcity [2,16,113]. The largest macropodid known from the Late Miocene is the sthenurine *Hadronomas puckridgi*, which has been estimated at approximately 73 kg body mass [114]. *Dorcopsoides fossilis*, from the same fossil locality and time period, is considerably lighter. This could be interpreted to imply niche partitioning between these two coeval browsers [115].

### 5.2. Functional morphology

*Dorcopsoides fossilis* is most consistently similar in limb morphology to low- to mid-geared extant macropodines, in particular, the dorcopsins and *Thylogale billardierii* (a forest and woodland wallaby from southeastern Australia). However, there are also similarities to higher-geared macropodines like *Notamacropus eugenii*. These characteristics are interpreted to imply that *Dd. fossilis* utilized its forelimbs for slow locomotion only, and that it was, in some respects, better equipped than its dorcopsin descendants to hop bipedally with power and efficiency. The sum of hindlimb observations suggests that *Dd. fossilis* was a generalized hopper, adapted for bursts of high-speed bipedal hopping but adept at lower-speed movement with frequent changes of direction. While otherwise similar to modern dorcopsins, and lacking the hindlimb proportions of long-distance hoppers, *Dd. fossilis* appears to have been able to powerfully flex its hip, knee and ankle joints to a degree similar to living mid-geared macropodines, such as *No. eugenii*. These conclusions are supported by the results of the PCA, which grouped *Dd. fossilis* with the low-geared taxa *Setonix brachyurus*, *Thylogale billardierii*, *Dorcopsulus vanheurni* and two species of *Dorcopsis*, while also showing some overlap with the mid-geared group, *Notamacropus rufogriseus*, *No. eugenii* and *Lagostrophus fasciatus*.

#### 5.2.1. Forelimb

The forelimb of *Dd. fossilis* is distinctly different from that of *Dendrolagus lumholtzi*, *Bettongia lesueur* and *Potorous tridactylus*, but is more similar to that of *Setonix brachyurus*. This suggests that *Dd. fossilis* was unspecialized in the use of its forelimbs, was not scansorial, arboreal or a habitual scratch-digger, and was predominantly bipedal at speed. Traits suggestive of flexibility in the metacarpal–phalangeal joints may be linked to occasional scratch-digging, or to grasping and manipulation of plant matter during feeding.

Differences in the morphology of the distal end of the humerus compared with that of lower-geared macropodids imply that *Dd. fossilis* was not adapted for fossorial activity. A shorter and less proximally pointed lateral supracondylar crest in *Dd. fossilis* suggests that the m. brachioradialis (elbow flexor), m. extensor carpi radialis, m. extensor carpi ulnaris, m. extensor digitorum communis, and m. extensor digitorum minimus (manual and digital extensors) [85,116] were not as well developed as in *S. brachyurus*, *B. lesueur* and *Po. tridactylus*. In these taxa, the lateral supracondylar crest is elongate and has a distinctly prominent proximal peak. A proximally extended origin for the m. brachioradialis on the pointed lateral supracondylar crest has been identified as improving the potential mechanical advantage by increasing in-lever length for the extensors [85]. This is likely associated with extension of the manus during fast quadrupedal locomotion in *S. brachyurus* [62], and with powerful, habitual digging for fungi and tubers in the case of the two potoroids [117,118]. This crest is slightly broader and more proximally pointed than in the three compared dorcopsins. *Dorcopsulus vanheurni* and at least two species of *Dorcopsis* are known to use their forelimbs during low- and mid-speed locomotion [62,70] and to consume some subterranean fungi [54], behaviour which necessitates scratch-digging. A smaller and less pointed lateral supracondylar crest in the living, scratch-digging dorcopsins suggests that *Dd. fossilis* engaged in more scratch-digging than living dorcopsins, or perhaps that it dug in harder substrate.

The shape of the distal humeral articulation of *Dd. fossilis* may demonstrate similarity in function to the humeri of living low- and mid-geared hoppers. In a study exploring the links between capitulum and trochlea relative size and shape and the nature of a marsupial’s forearm (distal forelimb) use, Jones *et al.* [119] linked a relatively larger and more rounded and convex capitulum and a smaller, shorter trochlea to forearm rotation and an arboreal habit, and a flatter capitulum and larger, wedge-shaped trochlea to a weight-bearing, pronated manus and a terrestrial habit. *Dorcopsoides fossilis* has a rounded capitulum paired with a fairly large, wedge-shaped trochlea, resembling that of compared species of *Notamacropus* [120], *Petrogale* Gray, 1837, [121] *Thylogale* Gray, 1837 [121] and *Dorcopsulus*. This suggests comparable use of the forearm by *Dd. fossilis* to these taxa, which regularly use their forelimbs for weight-bearing and have mixed-feeding and browsing diets [17,55,62]. The latter two scratch-dig for subterranean fungi [54].

There is no suggestion in the forelimb of *Dd. fossilis* of scansorial or arboreal adaptation. The trochlear notch on the ulna of *Dd. fossilis* is deeper than that of *De. lumholtzi*. A shallow trochlear notch is a feature of all species of *Dendrolagus*, probably providing scope for multiaxial movement. The deeper trochlear notch of ground-dwelling macropodines has been linked to a need for stability in the joint [85], a feature also present in *Ha. puckridgi*. The medial flexor fossa on the ulna is shallower and less elongate in *Dd. fossilis* than in *De. lumholtzi*, as well as in *S. brachyurus*, *Du. vanheurni* and the two potoroids. On the olecranon this fossa houses the insertion of the medial head of the m. triceps brachii, while its remaining area holds the partial origin of the m. flexor digitalis profundus, the major flexor of all five digits [85,105]. The muscle and its area of ulnar origin are notably more enlarged in arboreal and fossorial macropodines [85,116]. It is thus apparent that *Dd. fossilis* used its forelimb in a manner more typical of terrestrial macropodids.

There is further evidence in the ulna of *Dd. fossilis* that it was less fossorial than the potoroids. The olecranon of *Dd. fossilis* is moderately long relative to the height of the ulna at the trochlear notch, shorter than in *B. lesueur* and *Po. tridactylus* and similar to most macropodines, being relatively longer than in *De. lumholtzi*, *Do. luctuosa* and *Do. muelleri*. The olecranon holds the insertion of the mm. triceps, which are major extensors of the elbow joint. As the in-lever for the elbow extension system, its relative length is a measure of the extension power of the distal forelimb [122]. Fossorial animals typically have elongate olecranon processes to increase extension power [123], as manifested in the longer olecranons of the two potoroids. The mid-length olecranon of *Dd. fossilis* implies that its elbow required similar extension power to most macropodines. This reinforces the humeral evidence that *Dd. fossilis* used its forelimb for neither habitual digging nor high-speed locomotion.

The morphology of the radius of *Dd. fossilis* is interpreted as indicating an intermediate locomotive state, most similar in living macropodines to that of species of *Thylogale*, between the more specialized bipedality of the crown macropodins and the greater forelimb use during locomotion of *Du. vanheurni*, *Do. luctuosa*, *Do. muelleri* and *Po. tridactylus*. The gently craniocaudally compressed diaphysis of the proximal radius is similar in state to *T. billardierii* and the three compared dorcopsins, with the broad, flattened caudal area immediately distal to the radial tubercle likely housing a moderately well-developed partial origin of the m. flexor digitorum profundus [116]. The medial ridge along the radius, for the insertions of the m. supinator and m. pronator teres, are similarly developed to those of *M. fuliginosus*, *No. eugenii* and *T. billardierii*, suggesting little requirement to rotate the manus, as would be necessary in obligate quadrupedal locomotion, fossoriality or arboreality. The relative size and position of the radial tuberosity, to which inserts the major elbow flexor m. biceps brachii, is most comparable in *Dd. fossilis* to those of *No. eugenii*, *Du. vanheurni*, *Do. luctuosa* and *Do. muelleri*. This indicates similar elbow flexion requirements in these five species, with the same relative position of the tuberosity along the radius highlighting a similar in-lever versus out-lever length of the m. biceps brachii. It is most apparent in the radius of *Dd. fossilis* that the functional morphology of its forelimb was not identical to that of any one living species. It is instead, and perhaps unsurprisingly, intermediate in many respects and comparable to a range of macropodines of different ecomorphologies.

The distal facets of the metacarpals of *Dd. fossilis* are proximally extensive on both dorsal and palmar surfaces, most similar to those of *Du. vanheurni* and suggesting a high degree of digital flexibility in both flexion and extension. Additionally, the distal facets have a prominent palmar keel, most similar to that observed in *S. brachyurus* and *T. billardierii*. The keel separates and stabilizes the palmar sesamoids, which in turn serve to anchor and redirect the lines of pull of the flexor tendons, improving the efficacy of the flexors [124]. A larger keel suggests the presence of larger sesamoids, which would be required for action on larger and more powerful digital flexors. Unfortunately, the dietary habits and specifics of forelimb use of *Du. vanheurni* are not known, so the comparison is not particularly informative. Most of the metacarpal facets of *Dd. fossilis* are more proximally extensive than in *M. fuliginosus* and *No. eugenii*. These two species do not typically handle their food; *M. fuliginosus* does so rarely, typically apprehending food items directly with their incisors, while *No. eugenii* uses flat, opposing palms to hold food rather than grasping with their digits [125,126]. These behaviours are typical of crown macropodin taxa, but unlike other macropodids. For example, in *S. brachyurus* and *T. billardierii*, manual handling of food is more common and digital flexion is used regularly [125–127]. This may be interpreted to suggest the use of the digits to handle food by *Dd. fossilis*.

The presence of large collateral fossae on the distal metacarpals of *Dd. fossilis* runs counter to the evidence from the forelimb that the species did not undertake powerful flexion and extension of the manus. The shape and size of the collateral fossae are most similar in *S. brachyurus*, *T. billardierii*, *Du. vanheurni*, *Do. luctuosa* and *Do. muelleri.* The collateral ligaments stabilize the joint and help control its mobility [128], so larger tendons suggest a more mobile joint requiring greater stability. A need for stability in the metacarpal–phalangeal joints of a ground-dwelling kangaroo may be suggestive of greater forelimb weight-bearing.

The hypothesized sexual dimorphism in forelimb size and morphology in *Dd. fossilis* is consistent with that observed in extant sexually dimorphic macropodines. In these taxa males have greater body mass and positive allometry in the proportions and musculature of their forelimbs [129–131]. This is driven by male–male sexual selection via ‘boxing’ fights, where males stand up straight and pull, push and strike at the head and chest of their opponent [129,132]. Due to their gregariousness and propensity to form large, mixed-sex groups, larger-bodied, open-habitat macropodines show the strongest dimorphism [130,133]. Indeed, some large males of *Osphranter rufus* weigh more than twice the maximum weight of females [129].

The length of the humerus relative to that of the distal forelimb and the hindlimb increases with species size in male macropodines [130]. In all 15 macropodine species analysed in that study, males of a given species had longer forelimbs than did females with equivalent hindlimb length. Sexual dimorphism is here considered the most likely explanation for the size dimorphism present in the sample of adult humeri, ulnae and hamata of *Dd. fossilis* (figure 2). The muscles responsible for shoulder adduction and elbow and manual flexion were found to be highly dimorphic by mass in a study of *M. fuliginosus* [64]. The elements of *Dd. fossilis* hypothesized to be male all show considerably better-developed attachment and transmission areas, particularly those associated with adduction and flexion of the forelimb. Muscles performing these actions were identified as strongly sexually dimorphic in *M. fuliginosus* [131]. A longer and more raised pectoral crest in the ‘male’ humeral morph of *Dd. fossilis* would support a larger m. pectoralis, a major shoulder adductor [116]. A larger medial epicondyle on the humerus, medial flexor fossa on the ulna and palmar process on the hamatum would all facilitate better-developed manual flexor musculature (m. flexor carpi ulnaris and m. flexor digitorum profundus) [116]. This supports the notion that the perceived dimorphism is related to sex. As with many aspects of the biology of the species of *Dorcopsis* and *Dorcopsulus*, the degree of sexual dimorphism has not been described. Raw measurements from a generic review indicate slightly greater skull lengths in males than in females in each of the four species of *Dorcopsis*, but this was from samples of fewer than ten specimens per species [52]. Regardless, it seems likely that sexual dimorphism existed in *Dd. fossilis*, perhaps to a degree comparable with that of modern small- to mid-sized macropodines, such as species of *Thylogale* and *Petrogale*. It follows that they underwent male–male sexual selection via similar grappling and boxing fights for mating rights, behaviour noted in the related *Dorcopsis luctuosa* [132].

#### 5.2.2. Hindlimb

The pelvis of *Dd. fossilis* is similar to those of low- and -mid-geared macropodines, with a small number of features shared with the higher-geared macropodins. It is akin in general proportions to those of *Petrogale xanthopus*, *Thylogale billardierii*, *Du. vanheurni*, *Dorcopsis luctuosa* and *Do. muelleri*. The more craniodorsal placement of the large, rugose rectus tubercle, origin for the hip flexor m. rectus femoris, is similar in *Dd. fossilis* to that of *M. fuliginosus*, *No. eugenii*, *Pe. xanthopus* and *T. billardierii*. This would provide greater mechanical advantage for the in-lever, generating more powerful flexion of the hip. A deep iliac fossa in *Dd. fossilis* suggests a well-developed m. iliacus. A larger, deeper origin for this major hip flexor [87] than is present in the more quadrupedal *S. brachyurus*, *De. lumholtzi* and *Po. tridactylus* implies a greater ability to flex the hip joint and a more powerful hop.

As in the pelvis, the general proportions of the femur of *Dd. fossilis* are similar to those of lower-geared macropodids, but with some evidence from muscle attachments suggesting a more powerful and more efficient hop. The femur is similar in proportion to that of the low-geared and predominantly quadrupedal *S. brachyurus*, more gracile than the short and robust femora of higher-geared species like *M. fuliginosus* but more robust than in *De. lumholtzi*, *B. lesueur* and *Po. tridactylus*. A relatively proximally situated m. quadratus femoris insertion indicates more efficient locomotion and increased cursoriality (i.e. higher-gearing) when compared with *S. brachyurus*, *De. lumholtzi* and *Do. luctuosa*, with the proximal restriction of limb musculature increasing the mechanical work required for the same action but moving the distal limb further [134].

The proximal end of the femur is very similar in morphology to that of living dorcopsins in its articulation and muscle attachments. In the taxa examined for this study, a strongly medially projected lesser trochanter and a large, laterally projected proximolateral crest are unique to members of the Dorcopsini. The lack of specific knowledge of dorcopsin locomotory modes makes it difficult to grasp the likely functional significance of a more medially inserting m. iliopsoas (hip flexor) and a larger, more laterally inserting m. gluteus medius (hip extensor) [86,87] in the function of the hip joint. However, it is possible that they facilitate greater rotation.

The distal femoral epiphysis of *Dd. fossilis* is similar in proportions to all other species with which it was compared, with two exceptions. By comparison, *M. fuliginosus* has a taller, narrower epiphysis to support rapid and continuous action of the knee joint, whereas *De. lumholtzi* has a broader epiphysis to support the knee joint as the ankle rotates mediolaterally and weight is spread across a broader foot. The conservative nature of the knee joint in macropodines is apparent from the similarity across these comparisons.

The tibia and fibula of *Dd. fossilis* provide evidence of moderate hopping efficiency, intermediate between more plesiomorphic macropodoids on the one hand, and crown macropodins on the other. The short, strongly peaked form of the cnemial crest of *Dd. fossilis* is similar to that of all macropodines except for the species of *Dendrolagus* [2]. The relative length of the cnemial crest of *Dd. fossilis* is most similar to those of the extant dorcopsins, again intermediate between the shorter crests of higher-geared macropodines and the longer, weaker- or un-peaked crests of lower-geared macropodoids. Murray [82] demonstrated that a short and distally peaked cnemial crest is a derived feature of macropodines, with sthenurines having elongate cnemial crests blending smoothly into the distal diaphysis, also noted as present in *Ha. puckridgi* in our comparisons. The cnemial fossa houses the m. tibialis cranialis, with a proximally restricted muscle belly for this large pedal dorsiflexor [87] demonstrating some requirement to reduce the mechanical effort of distal hindlimb movement, implying higher gearing than *De. lumholtzi*, *Ha. puckridgi*, the two potoroids and *Hy. moschatus*.

The moderately deep peroneal groove on the distal end of the fibula of *Dd. fossilis* further emphasizes that it had similar locomotory adaptations to low- and mid-geared macropodoids. The peroneal groove is narrowest and shallowest in *M. fuliginosus*, *No. eugenii* and *Pe. xanthopus*, reflecting the reduction of podial dorsiflexor m. fibularis (peroneus) longus et brevis, which inserts to the base of the fifth metatarsal, and the digit V extensor and abductor m. extensor digitorum lateralis in these species [86,87,135]. This highlights the reliance on the major fourth pedal digit in these higher-geared species, and the relatively greater requirement for muscular action through, and lateral support by, the fifth digit in the lower-geared species [136].

The pes of *Dd. fossilis* is most similar to that of *T. billardierii* and living dorcopsins, though it also shares features with mid-geared taxa. The calcaneus illustrates this well: PCA of its proportions placed *Dd. fossilis* with both *T. billardierii* and *Du. vanheurni* (figure 10*a*), an affinity supported by comparisons of calcaneal morphology. Most *Dd. fossilis* calcanei plot within the low-geared group (figure 10*b*). There is likely some phylogenetic or allometric influence on the placement of *Lagostrophus fasciatus* close to the low-geared group, which influences the degree to which *Dd. fossilis* appears to overlap with the mid-geared group. Nonetheless, it is apparent from the spread of the group of *Dd. fossilis* towards the higher-geared taxa and the direction of the loadings that some specimens of *Dd. fossilis* had longer calcanei with narrower calcaneal heads and talar facets than the low-geared taxa in the analysis. These features—essentially a longer lever arm for the calcaneal tendon, a narrower sustentaculum tali, and narrower articulations with the tibia and fibula—can all be linked to more powerful plantar flexion of the ankle with less transverse rotation of the ankle joint [8,136]. The placement of the obligate-quadruped *Hy. moschatus* near to the high-geared group seems surprising, but is not indicative of shared function but rather a similarly narrow, elongate calcaneus. A similar convergent gracile calcaneus can be seen also in *Macrotis lagotis* (Reid, 137) [138]. This result highlights the role of phylogenetic proximity in analyses of this kind.

Other key morphological differences to the species of *Dorcopsis* are a more rounded sustentaculum tali in medial view and a narrower proximal section of the tuberosity where it meets the calcaneal head. Sthenurines also possess a squared (i.e. less rounded) sustentaculum tali in medial view, similar to that of species of *Dorcopsis*. A difference in the angle and curvature of the sustentaculum tali is likely related to the specific action of the tendon of the m. flexor digitorum profundus, an ancillary extensor of the ankle [86]. The presence of a curved sustentaculum tali in *Dd. fossilis* aligns the action of this tendon with those of non-dorcopsin macropodines and hints at slightly more rapid or efficient locomotion.

The relative gracility of the fourth metatarsal of macropodine kangaroos is considered an indicator of hopping ability. A more elongate metatarsal IV is generally associated with a faster, more efficient hop [17], with the elongated metatarsal increasing stride length and out-lever length [139,140]. The metatarsal IV of *Dd. fossilis* is more elongate than that of species of *Dorcopsis*, which may be interpreted to imply faster and more efficient locomotion. It has a length-to-width ratio similar to those of *No. eugenii*, *Pe. xanthopus* and *B. lesueur*. The elongation of the pes is augmented by the gracile proximal fourth phalanx and the cuboid of *Dd. fossilis*, the latter being unlike that of most compared species in almost as long as it is wide, rather than relatively considerably wider. Peculiarly, the gracility of the fourth metatarsal is less than that of *Du. vanheurni*, the locomotion and ecology of which is poorly documented, but which primarily inhabits dense lower and mid-montane forest [46,51] and is not known to be especially high-geared within the tribe. Unexplained characteristics such as this underline the need for increased ecomorphological study of living dorcopsins.

### 5.3. Palaeoecology

*Dorcopsoides fossilis* was a medium-sized wallaby that shared ecomorphological traits with extant dorcopsins and some more open-habitat macropodines. It has the crural structure of a moderately efficient bipedal hopper and a pedal morphology that points to a degree of flexibility. Overall similarity to the species of *Thylogale* may suggest that, in the manner of *T. thetis* and *T. billardierii* [141,142], *Dd. fossilis* sheltered at the fringe of dense habitats and moved into open areas to feed.

This palaeoecological interpretation is in keeping with the standing hypothesis for the Alcoota palaeoenvironment, and points to a significant difference with modern and other fossil dorcopsins. While the depositional environment of Alcoota itself was a shallow pond or lake-bed, faunal and palaeosol evidence from the Late Miocene demonstrates it to have been generally semi-arid, with similar annual precipitation to the modern day (100–450 mm), and with open woodlands dominating and gallery woodlands present [25,32,33]. This suggests *Dorcopsoides fossilis* to have been the most xeric-adapted of the dorcopsins. All other dorcopsins, including the fossil species *Watutia novaeguineae*, *Dorcopsoides buloloensis* and *Do. wintercookorum*, are associated with higher-rainfall environments [51,57,143]. The living dorcopsin that is closest to inhabiting similar woodlands to those present at Alcoota in the Late Miocene is arguably *Dorcopsis luctuosa*. This species chiefly utilizes primary and secondary rainforest in southern New Guinea; however, it has been recorded in drier tropical savannah and woodlands such as those near Port Moresby [47], which experience a significant dry season [144,145].

Lack of intimate knowledge of the locomotion and ecology of living dorcopsins limits our ability to compare and contrast the morphology of *Dorcopsoides fossilis* with its descendants and negatively impacts attempts to understand their evolutionary trajectories. The general differences between the ranges and habitat requirements of the species of *Dorcopsis* and *Dorcopsulus*, particularly in terms of altitude, are known and have been published [47–52]. However, the similarities and differences between species ecologies are practically unknown. Until the enigmatic New Guinean forest wallabies are appropriately investigated, we will be somewhat constrained in our ability to interpret the ecomorphology of *Dorcopsoides fossilis*.

### 5.4. Evolutionary implications

The limb morphology of *Dorcopsoides fossilis* is distinctly macropodine, demonstrating key affinities to the subfamily in most elements. Few similarities are shared with potoroids, and fewer are shared with the basal macropodid *Ngamaroo archeri. Ngamaroo archeri* has been described as possessing three derived features present in macropodines [94], two of which are noted present in *Dorcopsoides*: subequal-sized trochlea and capitulum on the humerus and a sinuous ventral ulnar margin. The ulnar morphology of *Ng. archeri* is otherwise distinctly potoroid-like, with only very slight recurve present in its ulnar diaphysis to link it to macropodines in this way. These two characteristics are supported by comparisons in this study and demonstrate that the macropodine body plan was well established by the Late Miocene. This bolsters the conclusions drawn from molecular divergence estimates that macropodines originated in the Middle Miocene (11–16 Ma) [18,19]. It also illustrates the important point that, while it might be the earliest-known macropodine, *Dorcopsoides fossilis* is sufficiently derived to indicate that it was a member of a radiation well underway during the Late Miocene, but currently concealed by a poor fossil record.

Modern dorcopsins may represent a return to a plesiomorphic forest-adapted state, rather than the retention of that ancestral state from Oligocene and Early Miocene macropodoids. This is at present still unclear; the fossil record of dorcopsins is so patchy and our understanding of their evolutionary relationships and past diversity is so poor that the evolutionary relationship of *Dorcopsoides* to other dorcopsins is uncertain. This secondary acquisition of seemingly plesiomorphic, forest-related traits is not so unusual within Marsupialia. Similar cases exist in the macropodine subtribe Dendrolagina and *Congruus kitcheneri* [146], both of which re-developed the ancestral tree-climbing habit [2,80], and the living phascoloricine dasyurids from New Guinea, which strongly exhibit primitive traits despite being nested well within Dasyuridae [147]. It is also possible that *Dorcopsoides fossilis* and its recently described congener *Dorcopsoides cowpatensis* [59] could well represent a divergent movement towards a drier habitat from an otherwise persistent mesic- and hydric-forest-associated palaeohistory for dorcopsins. *Dorcopsoides buloloensis* is a close relative of *Dorcopsoides fossilis* [59] and better knowledge of its palaeohabitat and postcranial morphology, both at present unknown, would aid in investigating the evolutionary trajectory of Dorcopsini.

## 6. Conclusion

The postcranial elements of *Dd. fossilis* described here represent an important step towards closing the knowledge gap between the derived, open-habitat-associated morphology of the Pliocene macropodines and the plesiomorphic Early and Middle Miocene macropodoids. *Dorcopsoides fossilis* has strongest affinities in its limb morphology to low-geared, plesiomorphic macropodines, with characteristics interpreted to imply some ability to quickly and efficiently cross open terrain. This is the earliest evidence of open-habitat adaptation in a macropodine kangaroo. These characteristics may suggest that living dorcopsins have secondarily moved into dense forests from an ancestral state, present in *Dd. fossilis*, of adaptation to scrubland and woodland. The lack of specific ecological knowledge conveyed by morphological resemblances between *Dorcopsoides* and modern species of *Dorcopsis* and *Dorcopsulus* underlines the need to expand study of the living dorcopsins.

## Data Availability

The datasets supporting this article have been uploaded as part of the electronic supplementary material. Datasets are available at Figshare [148]: 1, Extant comparative specimens; 2, Full measurements dataset; 3, Calcaneus PCA data; 4, Calcaneal and femoral mass estimates; 5, figures S1 and S2.
